# Adapting free energy perturbation simulations for large macrocyclic ligands: how to dissect contributions from direct binding and free ligand flexibility†
†Electronic supplementary information (ESI) available. See DOI: 10.1039/c9sc04705k


**DOI:** 10.1039/c9sc04705k

**Published:** 2020-01-22

**Authors:** Kerstin Wallraven, Fredrik L. Holmelin, Adrian Glas, Sven Hennig, Andrey I. Frolov, Tom N. Grossmann

**Affiliations:** a Department of Chemistry & Pharmaceutical Sciences , VU University Amsterdam , De Boelelaan 1083 , 1081 HV Amsterdam , The Netherlands . Email: t.n.grossmann@vu.nl; b Medicinal Chemistry, Research and Early Development Cardiovascular, Renal and Metabolism , BioPharmaceuticals R&D , AstraZeneca , Pepparedsleden 1, Mölndal , 431 83 , Sweden . Email: andrey.frolov@astrazeneca.com

## Abstract

Large and flexible ligands gain increasing interest in the development of bioactive agents. They challenge the applicability of computational ligand optimization strategies originally developed for small molecules. Free energy perturbation (FEP) is often used for predicting binding affinities of small molecule ligands, however, its use for more complex ligands remains limited. Herein, we report the structure-based design of peptide macrocycles targeting the protein binding site of human adaptor protein 14-3-3. We observe a surprisingly strong dependency of binding affinities on relatively small variations in substituent size. FEP was performed to rationalize observed trends. To account for insufficient convergence of FEP, restrained calculations were performed and complemented with extensive REST MD simulations of the free ligands. These calculations revealed that changes in affinity originate both from altered direct interactions and conformational changes of the free ligand. In addition, MD simulations provided the basis to rationalize unexpected trends in ligand lipophilicity. We also verified the anticipated interaction site and binding mode for one of the high affinity ligands by X-ray crystallography. The introduced fully-atomistic simulation protocol can be used to rationalize the development of structurally complex ligands which will support future ligand maturation efforts.

## Abstract 2

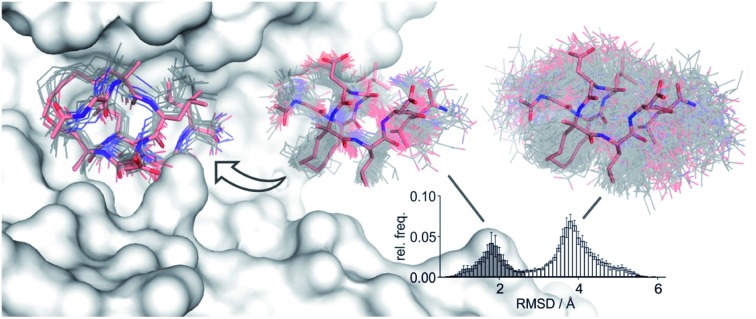
A combination of free energy perturbations and molecular dynamics simulations were applied to investigate large macrocyclic ligands and their receptor binding.

## Introduction

Selective ligands are the basis for most strategies aiming at the elucidation or modulation of biological processes.^1^ For protein targets with relatively flat surfaces, it still remains a major challenge to develop selective, high affinity ligands as traditional small molecular scaffolds usually require defined binding pockets.^2^ For such targets, more complex structures with large surface areas and more diverse conformational states represent a valuable source for ligands.^3^ In this respect, peptide-based scaffolds have proven useful, in particular when containing macrocyclic structures.^4^ Macrocyclization can reduce ligand flexibility thereby increasing target affinity and bioavailability.^5^ Such macrocycles still explore a large conformational space and a modulation of conformational constraints heavily impacts binding and physio-chemical properties such as lipophilicity.^6^,^7^ In addition to their overall high flexibility, the large number of individual interactions that contribute to binding complicate any affinity maturation process.^8^

Computational approaches allow to estimate binding affinities.^9^–^11^ In particular, the free energy perturbation (FEP) methodology based on molecular dynamics (MD) simulations proved useful for the quantification of relative binding free energies also providing mechanistic insights.^12^–^16^ Since FEP explicitly considers ligand and target flexibility,^17^ it tends to be more reliable than docking or molecular mechanics/Poisson–Boltzmann approaches.^9^,^18^ The FEP methodology is mainly applied for the characterization of small-molecule/protein complexes.^9^,^18^ The consideration of flexible, large and macrocyclic ligands on the other hand remains rather rare,^9^,^19^–^21^ since dynamic simulations of such systems often face convergence problems.^14^,^22^,^23^ For small molecules, restraining techniques have been applied to improve convergence of absolute binding free energy calculations^12^,^24^,^25^ also allowing the estimation of different contributions to the binding free energy, that are not readily accessible in experiments: *e.g.* conformational strain, contribution from electrostatic and van-der-Waals interactions.^12^ Yet, there is the need to establish and validate protocols of MD simulation-based methods for the rational design of compounds beyond small-molecular space.^3^

In particular, exploration of the vast conformational space of macrocyclic peptides is challenging, with some resent reports addressing this issue.^22^,^26^–^28^ Among those, force-field-based implicit solvent approaches proved to be very efficient in the exploration of conformational space.^27^,^28^ However, explicit solvent models are generally more accurate in predicting free energy quantities,^29^ but on the expense of increased computational cost. Various enhanced sampling techniques were proposed to accelerate explicit solvent simulations including Replica Exchange with Solute Tempering (REST) simulations,^30^ accelerated MD,^22^ metadynamics,^31^,^32^ adaptive umbrella sampling,^33^ and multiple simulations with diverse starting conformations analysed with Markov State Model.^26^ These methods allow to efficiently sample the conformational space of peptide macrocycles and to obtain the corresponding conformer populations. However, a general approach that includes this information into binding affinity predictions is lacking.

Here, we present the structure-based design of peptide macrocycles targeting the protein interaction site of human adaptor protein 14-3-3. Using a previously reported macrocyclic ligand as starting point,^34^ a small library of truncated derivatives with altered substitution pattern has been generated. FEP was performed and complemented with extensive REST MD simulations to rationalize the observed affinity trends. These calculations revealed that changes in affinity originate both from altered direct interactions and conformational changes of the free ligand. For one novel high affinity derivative a crystal structure in complex with 14-3-3 was obtained verifying the anticipated binding mode.

## Results

### Truncation and derivatization of macrocyclic peptides

**Fig. 1:**
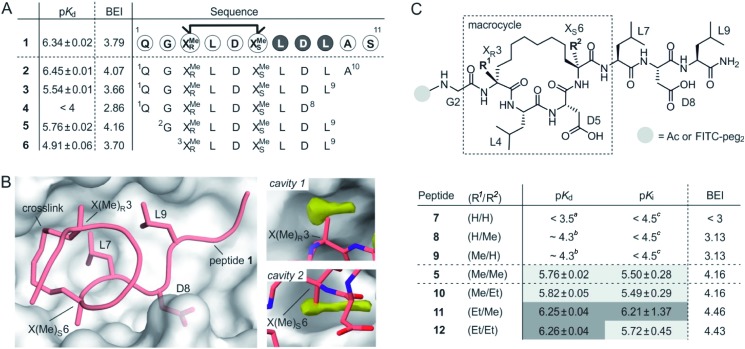
(A) Amino acid sequence of peptide **1** with dark circles highlighting the LDL hotspot motif, and truncation studies resulting in peptides **2–6** with corresponding p*K*_d_-values derived from direct FP and BEI-values. For affinity data see Fig. S1 and S2† (triplicates, errors account for 1*σ*); (B) crystal structure of **1** (light red, PDB ID ; 4n7y) in complex with 14-3-3 (grey) including hotspot amino acid side chains (aa **7–9**, LDL). Computational analysis of 14-3-3 surface reveals *cavity* 1 and 2 (yellow) in proximity to amino acids X(Me)_*R*_3 and X(Me)_*S*_6; (C) top: chemical structure of truncated peptide with variable substituents (R^1^, R^2^) and variable N-terminal modification; bottom: peptides with varying substitution pattern and corresponding p*K*_d_-, p*K*_i_- and BEI-values. p*K*_d_- and p*K*_i_-values are derived from direct FP and FP competition assays, respectively (triplicates, errors account for 1*σ*). ^*a*^Titration curves did not show significant change in signal (≤10%). Upper limit of p*K*_d_ was estimated based on highest protein concentration in titration; ^*b*^titration curve did not reach upper plateau. p*K*_d_ were calculated using the extrapolated upper plateau; ^*c*^titration curves did not show significant change in signal (≤10%). Upper limit of p*K*_i_ was estimated based on highest competitor concentration in titration.

Macrocyclic peptide **1** (Fig. 1A)^34^ binds to a class of highly related eukaryotic adaptor proteins called 14-3-3. It was originally derived from the pathogenic protein ExoS and proves efficient in inhibiting the interaction between ExoS and 14-3-3 proteins. The ExoS/14-3-3-interaction plays a crucial role in *Pseudomonas aeruginosa* infections which play an important role in hospital-acquired infections.^35^ Macrocyclic peptide **1** comprises 11 amino acids and harbors an *R*- and an *S*-configured α-methyl, α-alkyl amino acid at position 3 and 6, respectively (X(Me)_*R*_3 and X(Me)_*S*_6, Fig. 1B). Both amino acids are connected *via* their alkyl side chains forming an eight membered hydrocarbon crosslink. Notably, this hydrophobic crosslink contributes to binding by engaging in direct interactions with the target protein 14-3-3 and by stabilizing the bioactive conformation of the free ligand.^34^ Given the importance of the central macrocycle, we consider peptide **1** a good starting point for the structure-based design of smaller peptide ligands with high binding affinity.

Initially, we were interested to identify amino acid side chains in **1** that are crucial for 14-3-3 binding. These so-called hotspots, are defined as amino acid positions where variation to alanine results in considerably increased binding free energy (Δ*G* ≥ 2.0 kcal mol^–1^).^36^,^37^ Therefore, an alanine scan was performed by replacing each natural amino acid of **1**, except for glycine, sequentially to alanine. The resulting seven peptides were synthesized and N-terminally labeled with fluorescein isothiocyanate (FITC) for affinity testing in a direct fluorescence polarization (FP) assay. In analogy to previous studies, we used 14-3-3 isoform *ζ* (in the following, referred to as 14-3-3) for affinity measurments.^34^,^38^ In the FP assay, **1** served as the reference providing a dissociation constant (*K*_d_ = 0.46 μM, p*K*_d_ = 6.43) in the reported range.^34^ Alanine-variation of N-terminal amino acid Q1 and C-terminal S11 did not interfere with binding to 14-3-3 (Fig. 1A and S1†). This also holds true for central amino acids L4 and D5. We observed a severe loss of binding affinity when varying any of the three amino acids within the LDL motif (aa **7–9**, dark, Fig. 1A and S1†) thereby identifying these residues as hotspots. Encouraged by these findings, various N- and C-terminal truncations were tested to identify the minimal binding sequence of **1**. Truncation of the two C-terminal amino acids (A10 and S11) had only minor effects on binding affinity (p*K*_d_(**3**) = 5.54, Fig. 1A) while an additional deletion of L9 resulted in tremendously reduced affinity (p*K*_d_(**4**) < 4) which is in line with its hotspot character. Using peptide **3** as starting point, we tested N-terminal truncations indicating that removal of Q1 slightly improves binding (p*K*_d_(**5**) = 5.57) while the additional deletion of G2 considerably reduces affinity (p*K*_d_(**6**) = 4.91).

The binding efficiency index (BEI) is a useful measure when comparing a series of structurally related compounds to judge the importance of various groups.^39^ The BEI considers the dissociation constant (*K*_d_) of the target/ligand-complex in relation to the ligand's molecular weight (MW) (BEI = p*K*_d_/(MW × 10^–3^)).^40^ Among our truncation series, peptide **5** exhibits the highest binding efficiency (BEI = 4.2) thereby surpassing the 11-mer starting peptide **1** (BEI = 3.8). This renders **5** a good starting point for subsequent optimization aiming at increased binding affinity and efficiency. Due to the previously shown tolerance towards structural modifications,^34^ we pursued derivatization of the central macrocycle. Using the crystal structure of **1** in complex with 14-3-3 as structural basis, we searched for cavities in close proximity to the macrocycle (*l* ≥ 2 Å). The analysis of the 14-3-3 surface in this area, using an atomic probe placing approach,^41^ reveals two hydrophobic cavities (*cavity* 1 and 2, yellow, Fig. 1B).

*Cavity* 1 and 2 are only partially occupied by the two methyl groups of X(Me)_*R*_3 and X(Me)_*S*_6, respectively. Encouraged by this observation, we decided to test the effect of an ethyl group at the Cα of X(R^1^)_*R*_3 and X(R^2^)_*S*_6 (Fig. 1C). To probe the general influence of substitutions at those positions, we also included hydrogen bearing derivatives and assembled a panel of six macrocyclic peptides (**7–12**) with varying substitution patterns. Initially, these peptides were synthesized with an N-terminal FITC-label to determine their affinity for 14-3-3 using direct FP (p*K*_d_, Fig. 1C). Within this panel, only **7** (H/H) does not show detectable binding to 14-3-3 (p*K*_d_ < 3.5), while the two mono-methylated derivatives (**8** and **9**) exhibit low affinities (p*K*_d_ ∼ 4.3). Compared to **5** (Me/Me), all peptide derivatives with at least one H-substituent (**7–9**) experience a loss in binding affinity. Notably, peptides with ethyl substituents (**10–12**) show higher affinities than peptide **5** (Fig. 1C). Interestingly, ethyl modification at amino acid position 3 (X(Et)_*R*_3) results in a more pronounced affinity increase (Δp*K*_d_(**10**/**5**) = 0.06 *vs.* Δp*K*_d_(**11**/**5**) = 0.49). In addition, we do not observe an additive effect when introducing both ethyl groups (peptide **12**).

Due to their very similar molecular weight (MW = 1358–1412 g mol^–1^), differences in binding efficiency are mainly determined by the p*K*_d_-values rendering peptide **11** and **12** the most efficient binders (BEI = 4.46 and 4.43, respectively). To investigate potential effects of the fluorescent label on binding, we also performed FP competition experiments using N-terminally acetylated peptides. In these measurements, the 14-3-3 binding sequence of ExoS served as fluorescent tracer (Fig. S5†). Obtained IC_50_-values were used to calculate the corresponding p*K*_i_ values (Fig. 1C and Table S4†),^42^ which are generally in line with affinities derived from direct FP (p*K*_d_).

### Free energy perturbation calculations

Considering the small variations of substituent size relative to the macrocyclic ligand, we observe a strong dependency of binding affinities on the substitution pattern. To rationalize observed trends in binding affinities, FEP calculations were performed using the crystal structure of **1** in complex with 14-3-3 (PDB ID ; 4n7y) as starting model (see ESI for modelling details†). For our analysis, we decided to consider five different ligands: **7** (H/H), **9** (Me/H), **5** (Me/Me), **11** (Et/Me), **12** (Et/Et), covering the full diversity of our experimentally tested panel. The use of multiple ligands also allows to evaluate the convergence of FEP calculations by monitoring the hysteresis in thermodynamic cycles (Fig. S10†). While running conventional FEP simulations, we recognized insufficient convergence in particular for all edges with **7** (H/H), both in complex and in solvent (unbound) simulation legs. Unbound **7** (H/H) explores a broad conformational space as can be seen by time evolution of ligand RMSD (Fig. S11†). In addition, the conformation of bound **7** (H/H) drastically deviates from the X-ray-derived reference structure over time, such that the hydrophobic crosslink leaves the binding site (Fig. S12†). The latter, we interpret as initiation of an unbinding event, which is in line with the low measured binding affinity (p*K*_d_ < 3.5). Also, free energy estimates show significant drift over the entire simulation time, particularly for the edges with **7** (H/H) in solvent leg (Fig. S13†). These observations indicate insufficient sampling for the given simulation time (*t* = 20 ns per FEP leg), which is presumably inherent to the high flexibility and wide conformational space of the peptidic ligands.

**Fig. 2:**
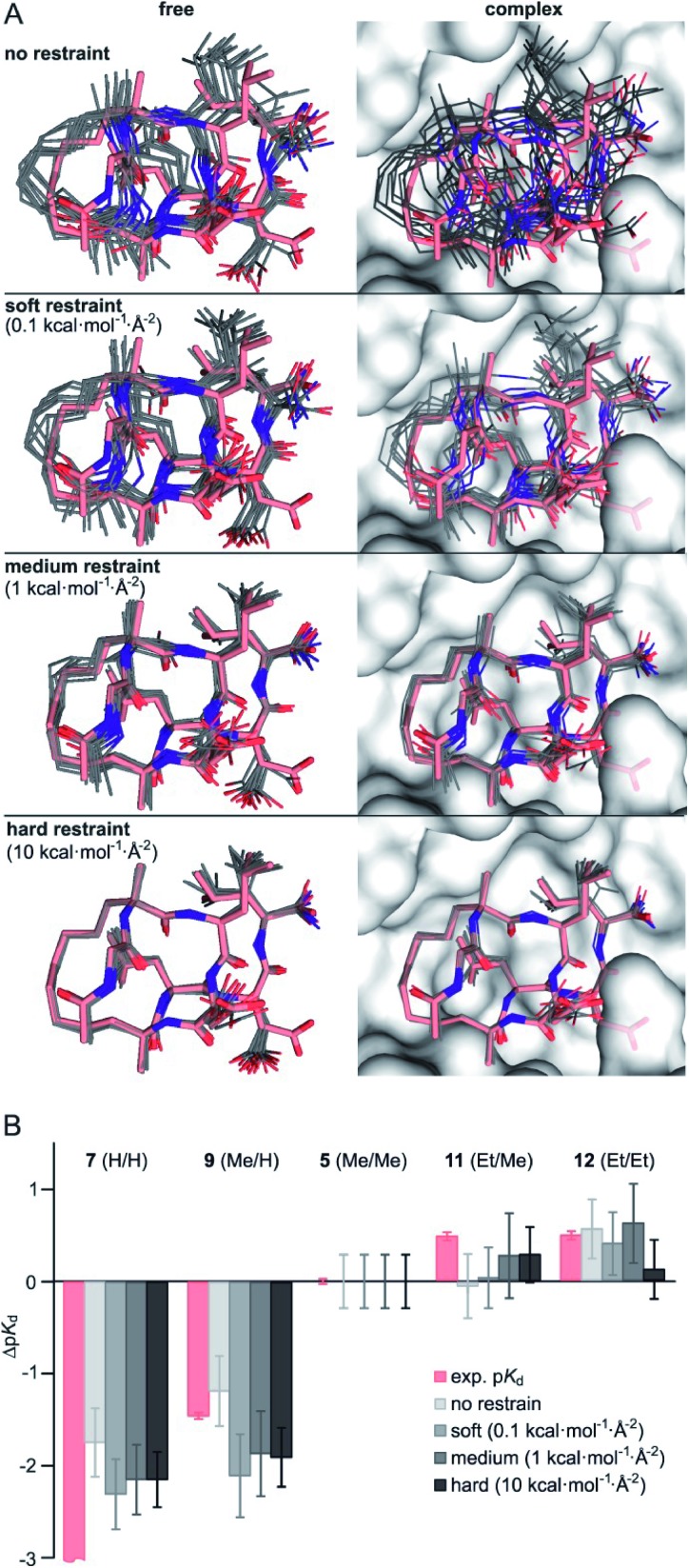
(A) Representative structures of FEP calculations for **5** (Me/Me, grey) with varying position restraints (force constants: 0 (none), 0.1 (soft), 1 (medium), 10 kcal mol^–1^ Å^–2^ (hard)). Trajectories are superimposed with **5** (Me/Me, light red) derived from crystal structure of **1** (aa **2–9**) in complex with 14-3-3 (grey surface, PDB ID ; 4n7y). Backbones of protein and ligand as well as the ligand crosslink are restrained to reference crystal structure of 14-3-3/**1**-complex; (B) Δp*K*_d_ values (Δp*K*_d_ = p*K*_d_(derivative) – p*K*_d_(**5**)) were experimentally determined by direct FP assays (light red) and calculated by FEP applying varying force constants of 0.1, 1 and 10 kcal mol^–1^ Å^–2^ (shades of grey, for values see Table S6†).

To minimize convergence problems and to estimate the contributions of direct interactions to binding free energies, FEP calculations with varying level of conformational restrains were performed applying three different restraining force constants (soft: 0.1, medium: 1, hard: 10 kcal mol^–1^ Å^–2^) to the ligand and to the protein backbone (Fig. 2A). With hard restraints, we achieved good convergence as ligands explore the same RMSD span over the simulation and running free energy estimates reaches the plateau quickly (Fig. S13†). As expected, representative simulation conformers (Fig. 2A) reveal a progressively reduced conformational diversity upon increasing the force constant both in the free and the bound state. This is also reflected by decreasing average RMSD-values (free: from 0.93 to 0.39 Å, bound: from 0.76 to 0.35 Å, Table S5†). FEP calculations provide Δp*K*_d_-values (relative to **5**, grey, Fig. 2B) which broadly recapitulate the experimental trends (light red).

Interestingly, although convergence improves at higher force constants, the different restraints provide similar Δp*K*_d_-values for a given peptide (Fig. 2B). Considering their varying degree of convergence, this indicates that both restrained and unrestrained FEP calculations mostly reflect contribution from direct protein–ligand interactions and solvation terms^43^, and do not capture conformational aspects.

Given the inherent accuracy limitations associated with FEP calculations,^9^ peptide **7** (H/H) and **9** (Me/H), as well as **5** (Me/Me), **11** (Et/Me) and **12** (Et/Et) can be considered to show similar predicted affinities (Fig. 2B) which is not fully in line with the experimental data. This and the fact that conformational aspects are presumably neglected in these FEP calculations encouraged further investigations regarding potential differences in the conformational aspects of the different free ligands.

### Molecular dynamics simulations of free ligands

**Fig. 3:**
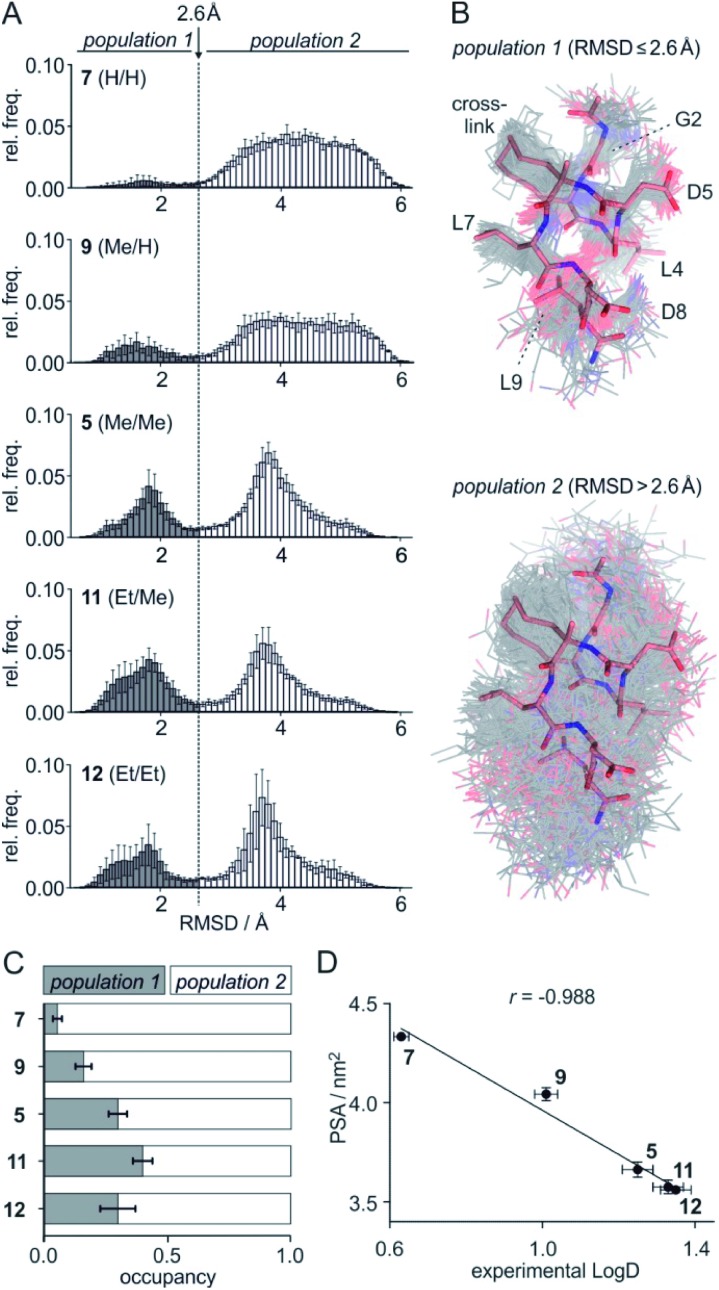
(A) Distribution of ligand RMSD derived from REST MD simulations (0.1 Å bin width) for selected peptides with varying substitution pattern (**7**, H/H; **9**, Me/H; **5**, Me/Me; **11**, Et/Me; **12**, Et/Et) shown as average over 5 blocks of 0.5 μs each (errors account for 1σ); (B) representative, simulated structures of **5** (Me/Me, grey) showing RMSD distributions for *population* 1 (RMSD ≤ 2.6 Å) and 2 (RMSD > 2.6 Å) superimposed with reference structure derived from peptide **1** (aa **2–9**, light red, PDB ID ; 4n7y); (C) relative distributions of *population* 1 and 2 based on REST MD calculations for selected peptides with varying substitution pattern; (D) correlation between PSA (polar surface area) and experimentally determined log *D*-values for peptides with varying substitution pattern including Pearson correlation coefficient (*r* = –0.988).

To assess the full conformational space of the free ligands in solution, we performed extensive Replica Exchange with Solute Tempering (REST) simulations.^30^ The full simulation time (*t* = 2.5 μs) was split into five blocks of 0.5 μs each to estimate statistical uncertainties. For each peptide, all five blocks show similar distributions of RMSD-values with respect to the crystal structure of **1** (Fig. S8†) indicating a consistent sampling of conformational space. Sufficient conformational sampling is also confirmed by time series of ligand RMSD revealing uniform distributions (Fig. S15†), and by the principal component analysis of the corresponding trajectories (Fig. S16–S18†). The latter shows that all ligands explore similar distinct conformational states. When looking at the torsion distribution of bonds within the macrocycle, we observe sampling of a similar conformational space for bonds that are remote from the alkyl substituents (Fig. S19 and S20†) indicating adequate sampling of macrocycle dihedral angles in the REST MD simulations. When plotting the frequency of RMSD-values, we observe a bimodal distribution of conformations for all peptides with a minimum around 2.6 Å (dashed line, Fig. 3A). Conformations with an RMSD ≤ 2.6 Å show good overlay with the reference structure (*population* 1, Fig. 3B top) while structures with an RMSD > 2.6 Å (*population* 2, Fig. 3B bottom) exhibit a diverse conformation pattern that differs substantially from the reference.

We reasoned that *population* 1 conformations provide an overall shape complementary to the binding site of 14-3-3 and are therefore more susceptible for binding than conformations from *population* 2. We recognize an increasing occupancy of *population* 1 with enlarging substituents (Fig. 3C): **7** (H/H, 6 ± 2%), **9** (Me/H, 16 ± 3%), **5** (Me/Me, 30 ± 4%) to **11** (Et/Me, 40 ± 4%). For peptide **12** (Et/Et, 30 ± 7%) though, we do not observe a further increase of *population* 1. Overall, alkylation of position X_*R*_3 and X_*S*_6 appears to promote *population* 1 presumably by restricting conformational freedom. This is in line with α-bisalkylated amino acids accessing a reduced range of *ψ* and *φ* dihedral angles when compared to their mono-substituted analogs.^44^ Most notably, the occupancy of *population* 1 and experimental p*K*_d_-values show a similar trend. Population of the bioactive conformation (*p*) has a direct effect on the apparent binding constant 
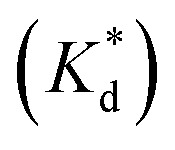
 *via* the following equation (see Section 1.8 in ESI for details†):1
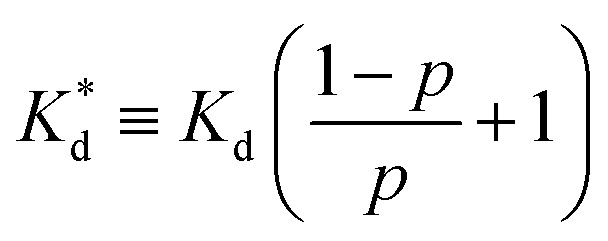
where *K*_d_ is the binding constant of the compound where the transition into the non-bioactive conformation is not permitted. The latter term accounts for the conformational equilibrium effect on the apparent binding affinity. The difference in binding affinities associated with the difference in populations of bioactive conformers between two ligands is then defined as:2
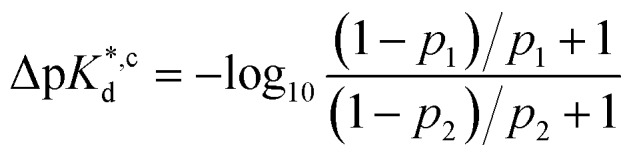



Considering the occupancy of *population* 1 for **7** (H/H) and **11** (Et/Me), one can estimate **7** (H/H) to be considerably less potent than **11** (Et/Me, Δp*K*_d_ ≈ 0.8. Table S7†).

Comparison of RMSD distribution from the 2.5 μs REST MD with the solvent legs of above presented 20 ns FEP simulations highlights the insufficient sampling of peptide conformation in FEP calculations (Fig. S14†). In FEP, ligands predominantly adopt *population* 1 conformations and barely access *population* 2. Clearly, short unbiased FEP simulations are not capable to sample accurately the ligand conformation space in bulk solvent in the case of studied, highly flexible peptides.

The lipophilicity of compounds is an important parameter determining their solubility and bioavailability.^45^ For this reason, we experimentally determined ligand log *D*-values reflecting the distribution of a compound between a hydrophobic and a hydrophilic phase. Here, we used a previously reported HPLC-based readout for log *D* determination.^46^ As expected, increased substituent size (H < Me < Et) is associated with higher log *D*-values (Table S9†): *e.g.* 0.63 (**7**, H/H) < 1.26 (**5**, Me/Me) < 1.35 (**12**, Et/Et). Surprisingly, log *D*-values nonlinearly increase with substituent size (Fig. S21†): *e.g.* the log *D* difference between **5** (Me/Me) and unsubstituted peptide **7** (H/H) (Δlog *D* = 0.63) is considerably larger than between ligands **12** (Et/Et) and **5** (Me/Me) (Δlog *D* = 0.09). To assess this behavior in more detail, log *P*-values were calculated based on 2D structures using a group contributions approach (Xlog *P*). These calculations result in an almost linear Xlog *P* increase with about 0.4 units per addition of a sp^3^-hybridized carbon (Fig. S21†)^47^ which is not in line with the experimental trend. Consequently, only a moderate correlation of calculated Xlog *P* values with our experimental log *D*-values is observed (Fig. S22,† *r* = 0.893). We hypothesized that this discrepancy originates from differences in populations and/or surface properties of 3D conformation states between the ligands.

To account for the 3D conformation effects on lipophilicity, we decided to compute the ensemble-averaged (dynamic) nonpolar and polar surface areas (NPSA and PSA, respectively) from above described REST MD simulations as those can be expected to determine affinity for the hydrophobic and aqueous phase, respectively. While calculated NPSA shows low correlation with experimental log *D*-values (Fig. S23,† *r* = 0.666), we observe an excellent correlation of the calculated PSA with log *D* (Fig. 3D, *r* = 0.988) suggesting the PSA having a dominating effect in our ligand panel. Analogous observations were reported for the correlation between cell permeability and linear combination of PSA and NPSA for a set of closely related peptides.^48^

Calculated surface area terms can be used in regression models for predicting ligand partitioning properties.^49^–^51^ Thus, we performed a multi-linear regression of measured log *D versus* PSA and NPSA descriptors providing calculated log *D*-values (log *D* = [0.27 × NPSA] – [0.82 × PSA] + 2.22). For these REST MD-derived parameters, calculated and experimental log *D*-values show an excellent correlation (Fig. S24,† *r* = 0.991). These observations highlight the importance of taking 3D conformational aspects of flexible macrocyclic molecules into consideration for computational predictions and rationalization of physicochemical properties.

### Co-crystallization of 14-3-3 with peptide **11** (Et/Me)

**Fig. 4:**
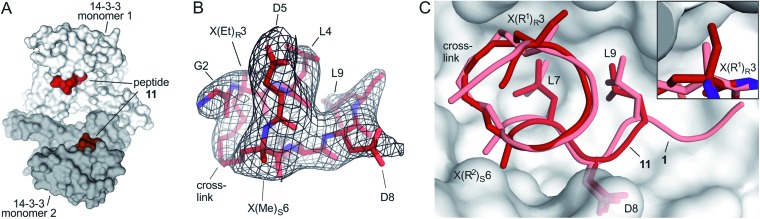
(A) Crystal structure of 14-3-3 dimer (light/dark grey surface) with each of the monomers occupied by one peptide **11** (Et/Me, red spheres, PDB ID ; 6rlz); (B) peptide **11** (red) enclosed by 2*F*_o_–*F*_c_ electron density map (black, contoured at *σ* = 1); (C) superimposed structures of peptide **11** (red, PDB ID ; 6rlz) and **1** (light red, PDB ID ; 4n7y) in complex with 14-3-3 showing the crosslink and side chains of hotspot residues (aa **7–9**, LDL). Close-up on *cavity* 1 occupied by the ethyl group of X(Et)_*R*_3 (peptide **11**, firebrick) and the methyl group of X(Me)_*R*_3 (**1**, light red).

Starting point for our simulations was the core of 11-mer peptide **1**. To investigate the binding mode of a truncated peptide in more detail, we aimed for a crystal structure of high affinity binder **11** (Et/Me) in complex with its target protein 14-3-3. Eventually, we were able to obtain crystals diffracting up to 3.7 Å (space group *P*6_4_, PDB ID ; 6rlz, Table S11†). The crystal structure harbors one 14-3-3 dimer in the asymmetric unit with each of the binding grooves occupied by **11** (Et/Me, Fig. 4A). For one peptide (chain C), the entire backbone and side chains are resolved. The electron density for peptide **11** (chain C) clearly shows the backbone as well as the location of side chains and crosslink (Fig. 4B). Superimposition of **11** (Et/Me) with starting peptide **1** reveals that both ligands bind to the same hydrophobic groove of 14-3-3. The two ligands show a close overlay (RMSD = 1.1 Å, Fig. 4C) in particular for the backbone and LDL-side chains (aa **7–9**). The additional ethyl group at amino acid X3 in **11** (Et/Me) points towards *cavity* 1 (Fig. 1B) thereby filling some of the partially unoccupied space observed for peptide **1**. Overall, the crystal structure verifies the anticipated binding mode of **11** and the initial motivation to vary the size of the α-methyl group.

## Discussion and conclusions

We report the structure-guided optimization of a macrocyclic peptide ligand targeting the protein binding groove of human adaptor protein 14-3-3. Our efforts resulted in a small ligand library containing macrocycle **11** with 23% reduced molecular weight and considerably increased binding efficiency compared to starting peptide **1**. Within our macrocycle library, we observed a surprisingly strong dependency of binding affinities on relatively small variations in substituent size (H, Me, Et) at the Cα atoms of the crosslinking amino acids.

To rationalize observed trends, we applied fully-atomistic FEP calculations, which however showed a lack of convergence for both structural and energetic parameters. To improve convergence, position restraints were implemented which allowed calculating the statistically converged contribution of direct interaction and solvation effects to binding. These calculations indicate that the observed affinity difference (Δp*K*_d_ ≈ 3) between high affinity binder **11** (Et/Me) and low affinity ligand **7** (H/H) appear to originate at least in part from differences in the direct interaction/solvation term (estimated Δp*K*_d_ ≈ 2).

To evaluate conformational aspects excluded by the implementation of restrains in FEP, we performed extensive REST MD simulations of the free ligands in water. These simulations reveal the existence of a conformational population similar to the bound state, whose occupation depends on the size of introduced substituents: larger substituents favor the bioactive conformation. *E.g.* the preference of ligand **11** (Et/Me) for the bioactive conformation, results in an additional gain in affinity of Δp*K*_d_ ≈ 0.8 when compared to ligand **7** (H/H). Considering the contribution of direct interactions (Δp*K*_d_ ≈ 2, based on FEP), our MD simulations suggest that conformational aspects account for *ca.* one third of the gain in binding affinity from **7** (H/H) to **11** (Et/Me). Notably, the here observed bimodal conformational behaviour cannot be expected to be a general feature of macrocyclic scaffolds, so that other ligand systems may require more elaborate analysis of conformational states. Also, REST MD was sufficient to obtain converged results here, however, other more enhanced sampling approaches could be applied if problems with convergence occur.^22^,^31^–^33^

Importantly, REST MD simulations of the free ligands also allowed to calculate dynamic polar surface areas which show an excellent correlation with experimental log *D*-values. The comparison with calculated Xlog *P*-values based on 2D structures, highlights the importance of considering 3D conformations. In addition, we were able to obtain a crystal structure of high affinity ligand **11** (Et/Me) in complex with 14-3-3 verifying the anticipated interaction site and binding mode. This is an important finding as it supports the relevance of the conformational restrains applied during FEP.

Analogously to classic FEP applications, a structurally well-characterized ligand–receptor complex is a prerequisite for the presented FEP/REST MD workflow, clearly complicating its application to ligands that adopt several binding modes in the same binding site^24^ or lack a sufficient degree of characterization. In addition, it is important to note that the restraining force constant applied for FEP calculations is an arbitrary parameter, which however could be debiased by explicitly calculating the reversible work required to introduce restraints potential.^12^,^24^ This requires reasonably converged simulations of unrestrained protein–ligand complexes, which for the present system appears to be rather challenging as we observe beginning of ligand unbinding in some of the unbiased simulations (Fig. S12†). Notably in the herein described system, calculated relative free energies only show low sensitivity towards the strength of applied restraints which indicates that the conformational bias affects ligands in a similar way. This is certainly an aspect that should be analyzed for more ligand/receptor pairs in the future.

Taken together, this is the first report of a fully-atomistic characterization of a complex between a large macrocyclic peptide and its protein binding partner, where the structure–activity relationship (SAR) is explained by dissecting different contributions into computed binding free energies. The combination of FEP and REST MD allows to separately quantify direct binding and conformational contributions to the binding free energy. This facilitated the rationalization of observed structure–affinity relationships. We believe this simulation protocol can be used to rationalize the development of structurally complex ligands, which increasingly gain attention as bioactive agents.

## Conflicts of interest

There are no conflicts to declare.

## Supplementary Material

Supplementary information

Supplementary information
