# Supplementary material for: Adapting free energy perturbation simulations for large macrocyclic ligands: how to dissect contributions from direct binding and free ligand flexibility
Source: Chem Sci. 2020 Jan 22;11(8):2269–76. doi: 10.1039/c9sc04705k (PMC7057854; doi:10.1039/c9sc04705k)
Supplement: Supplementary file 2 [file SC-011-C9SC04705K-s002.pdf]

Electronic Supplementary Material (ESI) for Chemical Science.
This journal is © The Royal Society of Chemistry 2020

SUPPORTING INFORMATION

Adapting free energy perturbation simulations for large macrocyclic ligands: How to dissect contributions from direct binding and free ligand flexibility

Kerstin Wallraven,[†] Fredrik L. Holmelin,[‡, ⊦] Adrian Glas,[†] Sven Hennig,[†] Andrey I. Frolov,*[, ‡] Tom N. Grossmann*[, †]

[†] Department of Chemistry & Pharmaceutical Sciences, VU University Amsterdam, De Boelelaan 1083, 1081 HV Amsterdam, The Netherlands

[‡] Medicinal Chemistry, Research and Early Development Cardiovascular, Renal and Metabolism, BioPharmaceuticals R&D, AstraZeneca, Gothenburg, Pepparedsleden 1, Mölndal, 431 83, Sweden

[*] Corresponding authors: A.I.F. (email: Andrey.frolov@astrazeneca.com), T.N.G. (email: t.n.grossmann@vu.nl)

Table of content

1. Methods 3

1.1. Building Block Synthesis and Characterization 3

1.2. Peptide Synthesis and Characterization 6

1.3. Protein Expression and Purification 7

1.4. Fluorescence Polarization Assays 8

1.5. Determination of LogD values 9

1.6. X-Ray Crystallography and Structure Determination 9

1.7. Computational Methods 10

1.8. Relation between ligand conformation equilibrium and ΔpKd 12

1.9. Relation between ligand distribution coefficient, PSA and NPSA. 14

2. Supporting Tables 15

3. Supporting Figures 21

4. References 39

1. Methods

1.1. Building Block Synthesis and Characterization

α-Ethyl containing amino acids were synthesized according to previously described protocols.[1]

Benzylprolinbenzophenon-Ni^{II}-homoalanine

To a solution of (*S*)/(*R*)-benzylprolinbenzophenone (1 eq) in MeOH (5 mL/mmol), Ni(II)nitrate hexahydrate (2 eq), rac-homoalanine (2 eq) and a solution of KOH in MeOH (8 eq, 0.1 mL MeOH per 1 mmol KOH) were added. The solution was heated up to 80 °C for 2 h, before quenching with acetic acid (8 eq). The reaction mixture was diluted with demineralized water (25 mL/mmol) and rested overnight, before the precipitate was isolated by filtration and washed with water. The remaining solid was dried under reduced pressure and purified via column chromatography on silica (5 % MeOH in DCM). The product was obtained as a red solid. Yields: (S): 61 %; (R): 84 %.

(*S*)

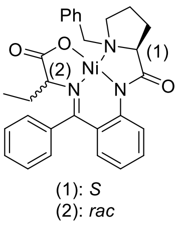

^{1}H NMR (500 MHz, DMSO-d6): δ = 8.37 (d, J = 7.1 Hz, 2H), 8.02 (dd, J = 8.7, 0.8 Hz, 1H), 7.55 (m, 4H), 7.37 (s, 2H), 7.15 (d, J = 7.4 Hz, 2H), 7.09 (s, 1H), 6.67 (d, J = 1.1 Hz, 1H), 6.54 (dd, J = 8.2, 1.5 Hz, 1H), 4.07 (d, J = 12.3 Hz, 1H), 3.63 – 3.53 (m, 3H), 3.35 (s, 2H), 2.48 (s, 2H), 2.19 (s, 2H), 1.87 – 1.76 (m, 1H), 1.60 – 1.48 (m, 1H), 1.25 (t, J = 7.4 Hz, 3H).

^{13}C-NMR (126 MHz, DMSO): δ = 180.11 (1C), 177.14 (1C), 169.69 (1C), 142.30 (1C), 134.63 (1C), 132.50 (2C), 131.32 (2C), 131.09 (2C), 129.41 (2C), 128.75 (1C), 128.62 (1C), 128.36 (1C), 128.17 (1C), 127.50 (1C), 127.08 (1C), 123.06 (1C), 119.88 (1C), 70.57 (1C), 69.55 (1C), 57.19 (1C), 30.28 (2C), 27.44 (1C), 23.15 (1C), 9.57 (1C).

(*R*)

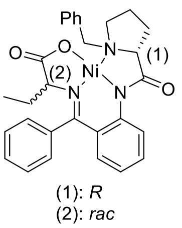

^{1}H NMR (500 MHz, DMSO-d6): δ = 8.35 (d, J = 7.2 Hz, 2H), 8.02 (dd, J = 8.7, 0.7 Hz, 1H), 7.57 (d, J = 22.4 Hz, 2H), 7.49 (d, J = 1.5 Hz, 2H), 7.36 (t, J = 7.7 Hz, 2H), 7.13 (d, J = 7.4 Hz, 2H), 7.08 (d, J = 1.6 Hz, 1H), 6.65 (s, 1H), 6.53 (dd, J = 8.2, 1.5 Hz, 1H), 4.06 (d, J = 12.3 Hz, 1H), 3.65 – 3.46 (m, 3H), 3.33 (d, J = 7.8 Hz, 2H), 2.46 (s, 2H), 2.17 (d, J = 9.7 Hz, 2H), 1.86 – 1.73 (m, 1H), 1.54 (m, 1H), 1.24 (t, J = 7.4 Hz, 3H).

^{13}C NMR (126 MHz, DMSO-d6): δ = 180.36 (1C), 177.39 (1C), 169.94 (1C), 142.55 (1C), 134.88 (1C), 134.03 (1C), 132.75 (2C), 131.57 (1C), 131.34 (2C), 129.65 (1C), 129.00 (1C), 128.86 (1C), 128.61 (1C), 128.42 (1C), 127.75 (1C), 127.32 (1C), 125.88 (1C), 123.31 (1C), 120.12 (1C), 70.83 (1C), 69.80 (1C), 62.80 (1C), 57.44 (1C), 30.53 (1C), 27.70 (1C), 23.40 (1C), 9.83 (1C).

(S)-Benzylprolinbenzophenon-Ni^{II}-2-ethyl-enoic acid

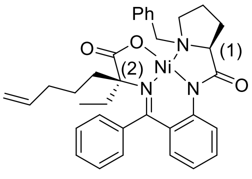

(1): *S*
(2): *S*

To a solution of (*S*)-benzylprolinbenzophenon-Ni^{II}-homoalanine (1 eq) in DMF (5 mL/mmol), freshly ground KOH (10 eq) was added under argon atmosphere at 0 °C. The reaction mixture was stirred for 20 min at 0 °C before warming up to room temperature and slowly adding 5-iodo-1-pentene (1.5 eq). After 7 h, the reaction was quenched by pouring it onto a cooled acetic acid solution (10 eq, 5 % in water). The resulting aqueous phase was extracted with DCM (3x). The organic phases were combined and dried over $MgSO_4$, before the solvent was removed under reduced pressure. The remaining crude contains a diastereomeric mixture, whereas (2) can be (*S*)- or (*R*)-configured. Separation of diastereomers was achieved via column chromatography on silica (EtOAc/PE, 4/1, v/v)). The (S)-configured main product was obtained as a red solid. Yield: 45 %.

^{1}H NMR (600 MHz, DMSO-d6): δ = 8.37 (d, J = 7.3 Hz, 2H), 7.85 (dd, J = 8.6, 1.2 Hz, 1H), 7.56 – 7.49 (m, 2H), 7.48 – 7.38 (m, 4H), 7.23 (t, J = 7.3 Hz, 1H), 7.09 (m, 2H), 6.63 (ddd, J = 8.3, 6.8, 1.3 Hz, 1H), 6.56 (dd, J = 8.4, 1.6 Hz, 1H), 5.90 (m, 1H), 5.06 (dd, J = 17.1, 1.9 Hz, 1H), 5.00 (d, J = 9.8 Hz, 1H), 3.95 (d, J = 12.3 Hz, 1H), 3.68 (d, J = 12.3 Hz, 1H), 3.54 (dd, J = 10.6, 6.1 Hz, 1H), 3.11 – 2.99 (m, 1H), 2.53 – 2.37 (m, 4H), 2.10 (q, J = 6.35 Hz, 2H), 2.06 – 1.93 (m, 3H), 1.53 – 1.32 (m, 3H), 1.22 (td, J = 13.3, 12.9, 4.0 Hz, 1H), 0.81 (t, J = 7.3 Hz, 3H).

^{13}C NMR (151 MHz, DMSO-d6): δ = 180.35 (1C), 180.00 (1C), 171.72 (1C), 141.73 (1C), 138.26 (1C), 136.22 (1C), 135.41 (1C), 132.64 (1C), 131.28 (1C), 130.69 (1C), 129.57 (2C), 128.51 (2C), 128.74 (2C), 128.06 (2C), 127.93 (1C), 127.22 (1C), 123.80 (1C), 120.08 (1C), 115.10 (1C), 81.45 (1C), 70.16 (1C), 63.31 (1C), 57.03 (1C), 37.58 (1C), 33.29 (1C), 33.22 (1C), 30.25 (1C), 24.18 (1C), 22.80 (1C), 8.55 (1C).

(*R*)-Benzylprolinbenzophenon-Ni^{II}-2-ethyl-enoic acid

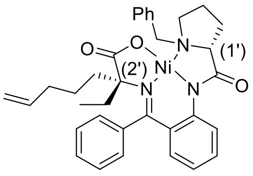

(1'): *R*
(2'): *R*

To a solution of (*R*)-benzylprolinbenzophenon-Ni^{II}-homoalanine (1 eq) in DMF (5 mL/mmol), freshly ground KOH (10 eq) was added under argon atmosphere at 0 °C. The reaction mixture was stirred for 20 min at 0 °C before warming up to room temperature and slowly adding 5-iodo-1-pentene (1.5 eq). After 7 h, the reaction was quenched by pouring it onto a cooled acetic acid solution (10 eq, 5 % in water). The resulting aqueous phase was extracted with DCM (3x). The organic phases were combined and dried over $MgSO_4$, before the solvent was removed under reduced pressure. The remaining crude contains a diastereomeric mixture, whereas (2’) can be (*R*)- or (*S*)-configured. Separation of diastereomers was achieved via column chromatography on silica (EtOAc/PE, 4/1, v/v)). The (*R*)-configured main product was obtained as a red solid. Yield: 35 %.

^{1}H NMR (600 MHz, DMSO-d6): δ = 8.37 (d, J = 7.5 Hz, 2H), 7.84 (d, J = 8.5 Hz, 1H), 7.56 – 7.49 (m, 2H), 7.47 – 7.34 (m, 5H), 7.23 (t, J = 7.4 Hz, 1H), 7.09 (m, 2H), 6.63 (t, J = 7.7 Hz, 1H), 6.56 (d, J = 8.2 Hz, 1H), 5.90 (m, 1H), 5.06 (d, J = 17.0 Hz, 1H), 5.00 (d, J = 10.1 Hz, 1H), 3.95 (d, J = 12.3 Hz, 1H), 3.69 (d, J = 12.3 Hz, 1H), 3.54 (dd, J = 10.6, 6.0 Hz, 1H), 2.50 (dt, J = 13.0, 8.9 Hz, 2H), 2.46 – 2.36 (m, 2H), 2.15 – 2.06 (m, 3H), 2.00 (m, 3H), 1.51 – 1.32 (m, 3H), 1.22 (td, J = 13.1, 3.9 Hz, 1H), 0.81 (t, J = 7.3 Hz, 3H).

^{13}C NMR (151 MHz, DMSO-d6): δ = 180.30 (1C), 179.93 (1C), 171.65 (1C), 141.64 (1C), 138.18 (1C), 136.14 (1C), 135.34 (1C), 132.56 (1C), 131.20 (2C), 130.62 (1C), 129.50 (1C), 128.44 (1C), 128.40 (2C), 127.99 (1C), 127.86 (1C), 127.84 (1C), 127.16 (1C), 127.14 (1C), 123.72 (1C), 120.02 (1C), 115.03 (1C), 70.09 (1C), 63.24 (1C), 56.95 (1C), 37.50 (1C), 34.74 (1C), 33.14 (1C), 32.75 (1C), 30.17 (1C), 24.11 (1C), 22.72 (1C), 8.46 (1C).

2-((((9H-fluoren-9-yl)methoxy)carbonyl)amino)-2-ethyl-enoic acid

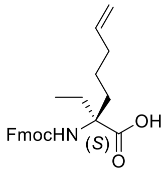

Fmoc-S_5^{Et}-OH

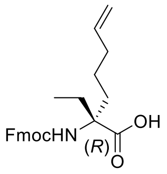

Fmoc-R_5^{Et}-OH

To a solution of benzylprolinbenzophenon-Ni^{II}-2-ethyl-eonic acid (1 eq) in MeOH (8 mL/mmol), conc. HCl (1 mL/mmol) was added, before heating up to 80 °C for 1 h. After cooling down to room temperature the solvent was removed under reduced pressure and the remaining solid was suspended in water before the solvent was removed again. The solid was suspended once more in water and extracted with DCM (3x), before the aqueous phase was adjusted to pH 10 with a saturated aqueous $NaHCO_3$ solution. An appropriate amount of dioxane and Fmoc-OSu (1.5 eq) were added to the solution. The reaction mixture was stirred at room temperature for 7 d under adding Fmoc-OSu (0.5 eq) every 24 h. Afterwards the reaction was diluted with demineralized water and acidified with a conc. HCl solution to pH 2-3. The aqueous phase has been extracted 3x with EtOAc, before combining and drying the organic phases over $MgSO_4$. The crude product was purified via column chromatography on silica (EtOAc/PE, 1/1, v/v + 0.1 % AcOH) and a subsequent PoraPak column (Waters®, 65 % ACN in water). The product was obtained as a white solid. Yields: Fmoc-S_5^{Et-}OH: 9 %; Fmoc-R_5^{Et-}OH: 8 %.

(*S*)

^{1}H NMR (600 MHz, DMSO-d6): δ = 12.69 (s, 1H), 7.89 (d, J = 7.5 Hz, 2H), 7.70 (d, J = 7.5 Hz, 2H), 7.41 (t, J = 7.4 Hz, 2H), 7.32 (td, J = 7.4, 1.1 Hz, 2H), 6.99 (s, 1H), 5.75 (ddt, J = 16.9, 10.0, 6.5 Hz, 1H), 4.99 (d, J = 17.2 Hz, 1H), 4.94 (d, J = 10.2 Hz, 1H), 4.28 (d, J = 7.9 Hz, 2H), 4.21 (t, J = 6.8 Hz, 1H), 1.97 (q, J = 7.2 Hz, 2H), 1.78 (m, 4H), 1.30 – 1.10 (m, 2H), 0.70 (t, J = 7.4 Hz, 3H).

^{13}C NMR (151 MHz, DMSO-d6): δ = 174.64 (1C), 154.04 (1C), 143.83 (2C), 140.72 (2C), 138.42 (1C), 127.60 (2C), 127.02 (2C), 125.19 (2C), 120.08 (2C), 114.90 (1C), 65.06 (1C), 62.14 (1C), 46.77 (1C), 33.20 (1C), 32.67 (1C), 26.50 (1C), 22.49 (1C), 7.85 (1C).

(*R*)

^{1}H NMR (600 MHz, DMSO-d6): δ = 12.67 (s, 1H), 7.89 (d, J = 7.5 Hz, 2H), 7.70 (d, J = 7.5 Hz, 2H), 7.41 (t, J = 7.4 Hz, 2H), 7.32 (td, J = 7.5, 1.1 Hz, 2H), 6.99 (s, 1H), 5.74 (ddd, J = 17.0, 11.7, 6.1 Hz, 1H), 4.99 (d, J = 17.2 Hz, 1H), 4.94 = (d, J = 9.8 Hz, 1H), 4.28 (d, J = 6.2 Hz, 2H), 4.21 (t, J = 6.8 Hz, 1H), 1.97 (q, J = 7.2 Hz, 2H), 1.78 (m, 4H), 1.29 – 1.11 (m, 2H), 0.70 (t, J = 7.4 Hz, 3H).

^{13}C NMR (151 MHz, DMSO-d6): δ = 174.62 (1C), 154.04 (1C), 143.82 (2C), 140.71 (2C), 138.43 (1C), 127.60 (2C), 127.02 (2C), 125.19 (2C), 120.08 (2C), 114.90 (1C), 65.05 (1C), 62.12 (1C), 46.76 (1C), 33.19 (1C), 32.66 (1C), 26.49 (1C), 22.48 (1C), 7.85 (1C).

1.2. Peptide Synthesis and Characterization

Peptides were synthesized manually on rink amide MBHA resin applying Fmoc-based solid-phase peptide synthesis. Chemicals were purchased from Iris Biotech, Merck, Okeanos, or Carl Roth and were used without further purification. Unless otherwise stated, reaction steps were carried out in syringe reactors at room temperature on an orbital shaker. Before synthesis, the resin was swollen in NMP for 2 h.

Fmoc removal

N-terminal Fmoc was removed using a solution of 25 % piperidine in NMP for 5min (2x).

Amino acid coupling

For the coupling of standard amino acids 4 eq were used with 4 eq of COMU ((1-Cyano-2-ethoxy-2-oxoethyliden-aminooxy)dimethylamino-morpholino-carbenium hexafluorophosphate), 4 eq of Oxyma Pure (Ethyl cyano (hydroxyimino) acetate) and 8 eq of DIPEA (*N*,*N*-Diisopropylethylamine) for 20 min. A second coupling was carried out using 4 eq of amino acid, 4 eq of PyBOP (Benzotriazol-1-yloxy)tripyrrolidino-phosphonium hexafluorophosphate) and 8 eq of NMM (*N*-Methylmorpholine) for 45 min. In case of non-natural amino acids 3 eq were used with 3 eq of PyBOP and 6 eq of DIPEA overnight. For amino acids coupled onto non-natural amino acids, triple couplings were performed with 6 eq amino acid, 6 eq COMU, 6 eq Oxyma and 12 eq DIPEA twice and with 6 eq amino acid, 6 eq PyBOP and 12 eq NMM once. All coupling solutions were prepared to yield a final concentration of 0.2 M regarding the resin loading and were discarded after reaction.

Capping

A capping step was applied using a solution of NMP/Ac_2O/DIPEA (10/1/1, v/v/v, 1 mL per 50 mg resin) for 5 min.

Ring-closing metathesis (RCM)

After finishing the peptide sequence non-natural α-methyl, α-alkenyl amino acids were cross-linked through ring-closing olefin metathesis. A solution of Grubbs 1st generation catalyst (4 mg/mL, 1 mL per 50 mg resin) in dry DCE was added to the resin under nitrogen stream for 2 h. This procedure was repeated four times in total, before the resin has been washed for 10 min with a DCM/DMSO solution (1/1, v/v) and with DCM only (3x).

Reduction of double bond

Reduction of the resulting double bond was performed at 55 °C for 1.5 h and 1000 rpm orbital shaking using a solution of 0.6 M TPSH and 1.2 M piperidine in NMP. After repeating this step four times in total, the resin was washed with NMP (3x), DCM (3x) and NMP (3x). Afterwards, Fmoc was removed to introduce *N*-terminal modifications.

***N*-terminal modification**

Acetylation of peptides was performed according to the capping protocol.
For fluorescence polarization binding assays, FITC (Fluorescein isothiocyanate isomer I) was attached as a fluorescence label via a Peg_2 (Fmoc-O2Oc-OH, 8-(9-Fluorenylmethyloxycarbonyl-amino)-3,6-dioxaoctanoic acid) linker. Peg_2 coupling follows the procedure for standard amino acids, while FITC was attached using 4 eq of fluorophore and 8 eq of DIPEA in NMP. The reaction precedes for 1 h and was performed twice in total.

Washing step

After each step, the resin was washed with NMP (3x), DCM (3x) and NMP (3x).

Cleavage, Purification and Characterization

Before final cleavage, peptides were washed with DCM (3x) and dried under reduced pressure. Afterwards, the resin was treated with a solution of TIPS/EDT/H_2O/TFA (1/2.5/2.5/94, v/v/v/v) for 1 h, twice. The solutions containing cleaved peptide were collected and TFA was evaporated under nitrogen stream. Ice-cold diethyl ether was added to the remaining solution, stored at – 20 °C for 30 min and centrifuged (10000 rpm, 4 °C, 10 min). The supernatant was removed and the remaining pellet dissolved in 30 % ACN (Acetonitrile). Afterwards the peptides were purified using a semi-preparative HPLC system with a Nucleodur C18 Gravity reverse-phase column (10 x 125 mm, 110 Å pore size, 5 µm particle size) using solvent A (H_2O + 0.1 % TFA) and solvent B (ACN + 0.1 % TFA). The concentration of FITC-labeled peptides was determined via FITC absorption at λ = 495 nm (ε = 77000 $M^{-1}\cdot cm^{-1}$) in 0.1 M Na_3PO_4 buffer (pH 8.5). For *N*-terminally acetylated peptides, concentrations were determined based on UV absorption at 210 nm (Table S1).

1.3. Protein Expression and Purification

Heterologous protein expression of 14-3-3ζ ΔC (aa 1 – 230) was carried out in *Escherichia coli* Rosetta (DE3) cells by transformation with pPROex HTb vector containing the corresponding gene. 200 mL LB medium were treated with 10 $\mu g\cdot mL^{-1}$ ampicillin before inoculation with transformed *E. coli* cells. The pre-culture was incubated at 37 °C and 170 rpm orbital shaking overnight. An appropriate volume pre-culture was used to inoculate 8 L of TB-medium (100 $\mu g\cdot mL^{-1}$ ampicillin) to yield an OD_{600} of 0.1. After growing the cell culture at 37 °C and 170 rpm orbital shaking to an OD_{600} of 0.6 – 0.8, protein expression was initiated by adding 0.5 mM IPTG. Incubation continued overnight at 25 °C and 150 rpm orbital shaking. Afterwards the cells were harvested by centrifugation at 4 °C and 4500 rpm for 20 min. The supernatant was discarded and DNase I and lysis buffer (50 mM tris(hydroxymethyl)aminomethane (TRIS) pH 8.0, 500 mM NaCl, 2 mM phenylmethylsulfonyl fluoride (PMSF), 2 mM, 2-mercaptoethanol) have been added to the remaining pellet before homogenizing by use of an ULTRA TURRAX dispenser. Afterwards the cells were lysed through a microfluidizer and the suspension was centrifuged at 4 °C and 8000 rpm for 30 min. The His-tagged protein was purified from the supernatant by affinity chromatography on nickel nitrilotriacetic acid (Ni-NTA) beads. Impurities were removed with a washing buffer (50 mM Tris pH 8.0, 500 mM NaCl, 5 % glycerol, 25 mM imidazole, 0.5 mM TCEP) and eventually His_6-tagged 14-3-3 has been eluted by an elution buffer (50 mM TRIS pH 8.0, 500 mM NaCl, 5 % glycerol, 25 mM imidazole, 0.5 mM tris(2-carboxyethyl)phosphine (TCEP)). The His-tag was removed by Tobacco Etch Virus (TEV) protease (0.05 mg protease per 1 mg protein) overnight at 4 °C. Afterwards 14-3-3ζ ΔC was concentrated and further purified by size exclusion chromatography (SEC) on an ÄKTA Pure system with a HiPrep 26/60 Sephacryl S-200 HR column using 20 mM 4-(2-hydroxyethyl)-1-piperazineethanesulfonic acid (HEPES, pH 7.4), 100 mM NaCl, 2 mM $MgCl_2$ and 1 mM 2-mercaptoethanol as buffer. The protein was concentrated by ultracentrifugation to 64 $mg\cdot mL^{-1}$ before flash frozen with liquid nitrogen for storage at -80 °C.

14-3-3ζ full-length His-tag was expressed according to the protocol described before. Here, *E. coli* BL21 gold (DE3) competent cells have been transformed with a pPROex HTb vector containing the corresponding gene. The protein was purified via affinity chromatography and subsequent size exclusion chromatography and has been used for further experiments with the His-tag remaining on the *N*-terminus.

1.4. Fluorescence Polarization Assays

Fluorescence polarization assay for determination of dissociation constants K_d

To determine the dissociation constants K_d of peptides towards 14-3-3ζ full length His-tag, FP (fluorescence polarization) assays was performed in triplicates. 100 µM DMSO stock solutions of *N*-terminally FITC-Peg labeled peptides were diluted with FP buffer (10 mM HEPES pH 7.4, 150 mM NaCl, 0.1 % Tween-20) to obtain a concentration of 40 nM. A 200 µM protein solution was diluted stepwise with FP buffer by a factor of 2.5 on a 384-multiwell plate (Corning, material no.: 4514). To each well containing 15 µL protein solution, 5 µL of 40 nM peptide solution was added to yield a final peptide concentration of 10 nM and protein concentrations ranging from 150 µM to 26 pM. The solutions were incubated for 15 min at room temperature, before the fluorescence polarization was measured using a Spark 20M plate reader (Tecan) with λ(ex)=485 nm and λ(em)=525 nm. To calculate K_d-values, GraphPad Prism® software was used applying a non-linear regression analysis of dose-response curves.

Competition fluorescence polarization assay for determination of inhibition constants IC_{50}

To determine the half maximal inhibitory concentration IC_{50} of peptides competing with ESp for binding of 14-3-3ζ full-length His-tag, FP-based competition assays were performed in triplicates. A 2.67 µM solution of protein in FP buffer (10 mM HEPES pH 7.4, 150 mM NaCl, 0.1 % Tween-20) was incubated with 66 nM FITC-peg$_2$ labeled ESp for 30 min. 10 mM DMSO stocks of *N*-terminally acetylated peptides were diluted with FP buffer to a concentration of 600 µM to be further diluted on a 384-multiwell plate (Corning, material no.: 4514) with a dilution factor of 1.5. 15 µL of pre-incubated protein/FITC-peg$_2$-ESp-complex were added to each well with 5 µL of acetylated peptide to obtain final Ac-peptide concentrations ranging from 150 µM to 101 nM and a 2 µM protein solution with 50 nM FITC-peg$_2$-ESp. The solutions were incubated for 1 h at room temperature, before the fluorescence polarization was measured using a Spark 20M plate reader (Tecan) with λ(ex)=485 nm and λ(em)=525 nm. To calculate IC_{50} values, GraphPad Prism® software was used applying a non-linear regression analysis of dose-response curves.

Calculation of inhibitory constant K_i

The inhibitory constant K_i was calculated based on K_d- and IC_{50}-values measured in direct FP and FP competition assays, respectively. Initially, K_i originates from inhibitory enzyme kinetics. Here, determination of K_i-values were performed according to a calculation strategy previously reported by Nikolovska-Coleska *et al.* allowing to describe inhibition of a protein without consideration of enzymatic activities.[2] Following equations (1) – (6) were used:

$$[P]_0^2 + (K_d + [L]_t - [P]_t) \cdot [P]_0 - [P]_t K_d \quad (1)$$

$$[P]_0 = -\left(\frac{K_d + [L]_t - [P]_t}{2}\right)\left(1 + \sqrt{1 + \frac{4^{[P]_t K_d}}{K_d + [L]_t - [P]_t{}^2}}\right) \quad (2)$$

$$[PL]_{50} = \frac{[PL]_0}{2} \quad (3)$$

$$[L]_{50} = [L]_{50} + [PL]_{50} \quad (4)$$

$$[I]_{50} = \mathrm{IC50} - [P]_t \left(+ K_d \frac{[PL]_{50}}{[L]_{50}}\right) = [PL]_{50} \quad (5)$$

$$K_i = \frac{[I]_{50}}{\frac{[L]_{50}}{K_d} + \frac{[P]_0}{K_d} + 1} \quad (6)$$

with $[P]_0$ = free protein concentration at 0 % inhibition in nM, K_d = dissociation constant of labeled tracer in direct FP assay in nM, $[L]_t$ = total concentration of labeled ligand in IC_{50} measurement, IC_{50} = half maximal inhibitory concentration of (unlabeled) inhibitor, $[P]_t$ = total protein concentration in direct FP in nM, $[PL]_{50}$ = concentration of protein/(labeled) ligand-complex at 50 % inhibition in nM, $[L]_{50}$ = concentration of the free labeled ligand at 50 % inhibition, $[I]_{50}$ = concentration of the free (unlabeled) inhibitor at 50 % inhibition.

1.5. Determination of LogD-values

LogD-values of *N*-terminally acetylated peptides were determined at pH 7.4 based on their retention time t_R applying an HPLC-UV method. Therefore, a calibration curve was established using reference compounds with previously measured LogD-values by octanol-water partitioning (supporting table 7, supporting figure 9A).[3–5] Retention times t_R for both reference compounds and peptides were obtained applying a gradient with a varying mixture of A (aqueous 50 mM ammonium acetate solution pH 7.4) and B (acetonitrile) as the mobile phase and a Nucleodur C18 Gravity reverse-phase column (10 x 125 mm, 110 Å pore size, 5 µm particle size) as the stationary phase with 1 mL/min flow rate. 0 min / 0 % B, 20 min / 95 % B, 30 min / 95 % B, 30.1 min / 0% B, 34.1 min / 0 % B was used as linear gradient. 10 mM DMSO stocks of reference compounds and peptides were prepared and diluted 1:5 with A. 5 µL of peptide solutions and between 2 and 50 µL of reference compound solutions were injected to the HPLC. UV-detection was performed at λ=254 nm. All retention times t_R were measured in triplicates and t_R-values for peptides were determined using equation (7) (supporting tables S8, S9, supporting figure S9A, S9B):

$$\text{LogD} = 0.5934 \cdot t_R - 6.3225 \qquad (7).$$

1.6. X-Ray Crystallography and Structure Determination

For co-crystallization, purified 14-3-3ζ ΔC (aa 1 – 230) was diluted to a final concentration of 22 mg/mL using complexation buffer (20 mM HEPES pH 7.5, 100 mM NaCl, 2 mM $MgCl_2$, 1 mM β-mercapoethanol). A DMSO stock solution of peptide **11** (Et/Me) was added to yield a molecular ration of 1:2 with a final DMSO concentration of 1 %. The protein/peptide-solution was incubated on ice for 3 h to reach equilibrium. Initial screens were performed in 96 well plate (TTP Labtech) applying the sitting-drop method at 4 °C with 70 µL reservoir buffer and by mixing 0.1 µL complex solution with 0.1 µL reservoir solution. JCSG Core I and JCSG Core IV (Qiagen) were used in screening for suitable crystallization conditions. Crystal growth was observed after 7 d at 0.2 M Mg formate with 20 % PEG 3350. This condition was further optimized in a 24 well plate (0.2 M Mg formate pH 7-8.5, 16-26% PEG 3350) employing the hanging-drop vapor diffusion method. Obtained crystals were fished and transferred to cryogenic condition (10 % glycerol) before snap frozen in liquid nitrogen. Diffraction data up to a resolution of 3.7 Å was collected at I24 beamline at the DLS (Diamond Light Source) in Oxfordshire, UK. XDS software package was applied for crystallographic analysis of data, whereas PHASER was used for molecular replacement and REFMAC5 (CCP4i software) for refinements. Model building has been carried out with COOT. Detailed statistics of data collection and refinements are shown in supporting Table S11.

1.7. Computational Methods

1.7.1 Molecular modelling details

The molecular modelling was performed using Schrödinger Suite 2017.3 and 2018.1 [Schrödinger Suite 2018; Schrödinger L. L. C.: New York, NY, 2018.]. The starting structures for the simulations were obtained from PDB structure 4n7y chain C. We manually implemented required truncations or addition/removal of methyl group to obtain the initial structures of studied ligands. We used the Protein Preparation Wizard to prepare the protein structure. The protonation states were assigned assuming a pH of 7.0. All crystallographic waters were included in simulations. The protein/ligand-complex and free ligand systems were hydrated in an orthorhombic simulation box with the minimum distance to the nearest solute atom (buffer width) of 5 Å for the complex simulations and 10 Å for the free ligand simulations. Protein and ligands were modeled with OPLS2.1 force field[6] with SPC water model,[7] the missing torsions at the X_R3 and X_S6 residues were parameterized using QM torsion profiles of corresponding fragments as implemented in the default force field builder of the Schrödinger suit. We applied the hydrogen mass repartitioning to increase the simulation time step.

1.7.2 MD simulation details

Simulations were done under periodic boundary conditions. The systems were equilibrated using the default Desmond MD relaxation protocol,[8] which includes 100 ps simulation at 10 K and 50 kcal·mol^{-1}·Å^2 harmonic restraining force on solute heavy atoms with Brownian dynamics and dual time step integrator with 1 and 3 ps time steps for bonded/short-range and long-range non-bonded interactions, correspondingly. It followed by 12 ps simulation with the Verlet integrator with Berendsen thermostat (10K, τ = 0.1 ps) and randomization of velocities every 1 ps. Then, 12 ps simulation in NPT ensemble with Berendsen barostat (1 bar, τ = 50 ps) and thermostat (τ = 0.1 ps). It followed by 24 ps simulation in NPT ensemble with Berendsen barostat (1 bar, τ = 50 ps) and thermostat (300K, τ = 0.1 ps) with restraints and then by 240 ps without restraints. Production simulation was run for 20 ns using the dual time step Verlet integrator with 4 and 6 fs time steps, Martyna-Tobias-Klein constant temperature (300 K, τ = 1 ps) and pressure algorithm (1bar, τ = 2 ps) and [Desmond User's Guide Desmond Version 3.0 /Document Version 0.5.3 D. E. Shaw Research 1 April 2011].

1.7.3 FEP calculation details

We utilized 12 λ windows for all the FEP calculations. We used the FEP/REST scheme[9] where the replica exchange between neighboring λ windows were attempted every 1.2 ps. No solute „hot region" was utilized in the FEP simulations. Trajectory frames were stored each 238 ps that resulted in 85 structures for each simulation. The Bennett acceptance ratio method (BAR)[10] was used to calculate the free energy differences. Error bars were estimated for each free energy value using both bootstrapping and the Bennett analytical error prediction. The free energies and the error bars were calculated using the cycle closure algorithm described elsewhere.[9] In the case of restrained FEP calculations we applied harmonic restraining potentials for heavy-atom atomic position deviation from the corresponding X-ray reference in X, Y, Z directions to protein backbone excluding oxygen atoms and N and O of the *C*-terminal amides, to the ligand heavy atoms excluding the substituents at X_R3 and X_S6 and side chains beyond β-carbon atoms (but keeping the crosslink atoms restrained).

1.7.4 REST MD simulation of free ligands in water

We utilized REST algorithm [10.1039/B509983H] with 8 replica with targeted probability of replica exchange of 0.15 and linear ladder scheme. Each replica was run for 2.5 µs and the replica exchange between neighboring replica was attempted every 1.2 ps, such that each replica could exchange only with one neighbor at a time and the neighbor was chosen randomly. To check the quality of simulations the

actual exchange probabilities between neighboring windows were calculated and were not lower than 10 %. Trajectory frames were stored each 100 ps that resulted in 2500 structures for each simulation.

1.7.5 RMSD calculations

In the case of simulation for complexes we overlaid trajectory frames by protein backbone on the reference X-ray structure and the RMSD of the heavy-atoms of the ligand (excluding the varied alkyl substituents at X_R3 and X_S6 and the symmetric oxygens of the carboxylic group sidechains) were calculated with respect to the reference X-ray structure. For this purposes we chose simulations with fully coupled **7** (H/H) and **5** (Me/Me) ligands from **7** (H/H) – **9** (Me/H) and **5** (Me/Me) – **11** (Et/Me) edges, correspondingly.

1.7.6 NPSA and PSA calculations

NPSA and PSA calculations were performed using the Gromacs 2016.04 utility "gmx sasa".[11] The desmond trajectories were converted to the xtc format and the corresponding gromacs topologies were generated with ffconv.py tool.[12] The PSA and NPSA algorithm implemented in Gromacs is described elsewhere.[13]

1.7.7 Principal component analysis (PCA) of the trajectories

PCA in the Cartesian space was performed using the Gromacs 2016.04 utilities "gmx covar" and "gmx anaeig".

1.8. Relation between ligand conformation equilibrium and ΔpK_d

We consider a reversible one-step binding equilibrium between a ligand and protein (P) forming complex (PL), where the ligand coexists in two conformations, one is bioactive (L), another is non-bioactive (L*). The transformation scheme can be written as follows:

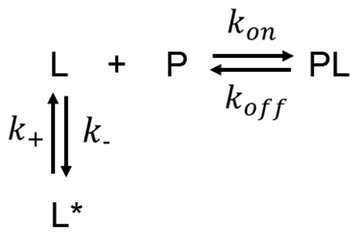

Where k denotes the corresponding reaction rate constants.

When equilibrium is reached, the following equations hold true:

$$\frac{d[PL]}{dt} = \frac{d[P]}{dt} \tag{8}$$

where $[X]$ denotes equilibrium concentration of species X, t denotes time, resulting in:

$$k_{on}[L][P] = k_{off}[PL] \tag{9}$$

$$\frac{\mathrm{k}_{off}}{\mathrm{k}_{\mathrm{on}}} = \frac{[\mathrm{L}][\mathrm{P}]}{[\mathrm{PL}]} \tag{10}$$

and:

$$\frac{d[L]}{dt} = \frac{d[L^*]}{dt} + \frac{d[PL]}{dt} \tag{11}$$

$$k_{off}[PL] + k_+[L^*] = k_-[L] + k_{on}[L][P] \tag{12}$$

Using Eq. 9, we get:

$$\frac{\mathrm{k}_-}{\mathrm{k}_+} = \frac{[L^*]}{[\mathrm{L}]} \equiv K_L \tag{13}$$

whereas K_L is the thermodynamic constant determining the ratio of concentrations of non-bioactive and bioactive ligand conformations. It can be written also as:

$$K_L = \frac{1 - p}{p} \tag{14}$$

where p is the population of bioactive conformer.

Ligand mass conservation gives us:

$$C_L = [PL] + [L*] + [L] \tag{15}$$

From Eq. 13 and Eq. 15 we can express the ligand equilibrium concentration as:

$$[\mathrm{L}] = \frac{\mathrm{C_L} - [\mathrm{PL}]}{\mathrm{K_L} + 1} \tag{16}$$

Protein mass conservation gives us:

$$\mathrm{C_P} = [\mathrm{PL}] + [\mathrm{P}] \tag{17}$$

From Eq. 10, 16, 17 we get:

$$\frac{\mathrm{k_{off}}}{\mathrm{k_{on}}}(\mathrm{K_L} + 1) = \frac{(\mathrm{C_L} - [\mathrm{PL}])(\mathrm{C_P} - [\mathrm{PL}])}{[\mathrm{PL}]} \tag{18}$$

When population of bioactive conformation becomes 100 %, $\mathrm{K_L} \rightarrow 0$, we recapitulate the regular single-step binding equilibrium with thermodynamic dissociation constant K_d:

$$K_d \equiv \frac{\mathrm{k_{off}}}{\mathrm{k_{on}}} = \frac{(\mathrm{C_L} - [\mathrm{PL}])(\mathrm{C_P} - [\mathrm{PL}])}{[\mathrm{PL}]} \tag{19}$$

In biophysics experiments, where the concentration of protein-ligand complex in equilibrium is the variable and population of bioactive conformation is not 100 %, the system will appear as a single-step binding equilibrium with effectively reduced (apparent) binding constant K_d*(compare Eq. 18, 19):

$$\mathrm{K_d^*} \equiv \mathrm{K_d}(\mathrm{K_L} + 1) \tag{20}$$

Changing the population of the bioactive conformation affects the apparent binding constant. Consider system 1 and system 2, which differ only in their equibrilium between bioactive and non-bioactive conformation: we assume that binding to the protein of the bioactive conformation is not affected, therefore K_d stays constant. Consequently, the ratio of apparent constants can be expressed as follows:

$$\frac{K_d^{*,1}}{K_d^{*,2}} = \frac{K_L^1 + 1}{K_L^2 + 1} \tag{21}$$

$$\Delta pK_d^{*,c} = -log_{10}\frac{K_L^1 + 1}{K_L^2 + 1} \tag{22}$$

Where the *c* superscript denotes that the difference is associated to difference in conformation equilibria.

In the limiting case, where we assume that all conformations are transformed into bioactive form ($\mathrm{K_L^2} \rightarrow 0$), we get:

$$\Delta pK_d^{*,c} = -log_{10}(K_L + 1) \tag{23}$$

As an example, we estimated the difference in pK_d between **11** (Et/Me) and **7** (H/H) ligands originating solely from conformational aspects as:

$$\Delta pK_d^{*,c}(H/H \rightarrow Et/Me) = -log_{10}\frac{60/40 + 1}{94/6 + 1} \approx 0.82 \tag{24}$$

1.9. Relation between ligand distribution coefficient, PSA and NPSA.

We use logD to describe the distribution of a ligand between two liquid phases: octanol and buffer (pH 7.4). The ligand distribution coefficient LogD is determined by the difference of excess chemical potentials (or equivalently, solvation free energies[14]) in the two phases:

$$\frac{RT\ logD}{2.303} = \Delta G_{aq}^{solv} - \Delta G_{oct}^{solv} \tag{25}$$

Solvation free energy is often approximated in a form of Poisson-Boltzmann expression supplemented with empirical contribution proportional to solvent accessible surface area (SASA) of the compound (PBSA) [https://doi.org/10.1021/ct050097l].

$$\Delta G_{aq}^{solv} = \alpha_{aq}\mathrm{SASA}_{aq} + \beta_{aq} + \Delta G_{aq}^{elec} \tag{26}$$

The latter SASA linear term is an attempt to describe the free energy of creating the cavity in the solvent, van-der-Waals interaction with solvent, while the Poisson-Boltzmann term captures the solvation terms originating from electrostatic interactions between ligand and solvent. Electrostatic solvation energy depends on the non-uniform electrostatic potential around the molecule. It is sensitive to group ionizations, dipole moments and their mutual arrangement in the molecule. However, for the set of ligands in the study, which differ only by small non-polar substituents, we assume that the electrostatic contribution is proportional to the accessible polar surface area (PSA).

$$\Delta G_{aq}^{elec} = \gamma_{aq}\mathrm{PSA}_{aq} + \sigma_{aq} \tag{27}$$

Using the expression $SASA = PSA + NPSA$, where NPSA denotes the non-polar surface area and combining eq. 25, 26, 27 we get:

$$\begin{aligned}\frac{RT\ logD}{2.303} = {} & (\alpha_{aq} + \gamma_{aq})\mathrm{PSA}_{aq} + \alpha_{aq}\mathrm{NPSA}_{aq} + \beta_{aq} + \sigma_{aq} \\ & - \left((\alpha_{oct} + \gamma_{oct})\mathrm{PSA}_{\mathrm{oct}} + \alpha_{oct}N\mathrm{PSA}_{\mathrm{oct}} + \beta_{oct} + \sigma_{oct}\right)\end{aligned} \tag{28}$$

Assuming that the PSA and NPSA remains the same for two solvents, which implicitly means that ligands explore similar conformation space in both solvents, we get the following:

$$\frac{RT\ logD}{2.303} = (\alpha_{aq} + \gamma_{aq} - \alpha_{oct} - \gamma_{oct})\mathrm{PSA} + (\alpha_{wat} - \alpha_{oct})\mathrm{NPSA} + \beta_{aq} - \beta_{oct} + \sigma_{aq} - \sigma_{oct}$$

$$\frac{RT\ logD}{2.303} = \mathrm{a} \cdot \mathrm{PSA} + \mathrm{b} \cdot \mathrm{NPSA} + \mathrm{c} \tag{29}$$

Eq. 28 indicates that there should be linear dependence of measured distribution coefficient, PSA and NPSA. We appreciate that the number of assumptions is large and the relation likely should work only for a set of congeneric series with identical ionization states. However, this relation gives us a guide of what type of correlation to expect between measured lipophilicity and SAs calculated in MD simulations.

2. Supporting Tables

Table S1: Detailed overview of all synthesized peptides. Retention times t_R were measured by HPLC and m/z ratios were obtained from mass spectrometric analysis by HPLC-coupled mass spectrometer.

Peptide	*N*-Term. mod.[a]	Sequence	HPLC Grad[b]	HPLC t_R / min	m / z (calc.)[c]	m / z (found)
**1**	Ac	QG-R_5^{Me}-LD-S_5^{Me}-LDLAS	1	12.68	1224.68	1224.6 $[M+H]^+$
	F	QG-R_5^{Me}-LD-S_5^{Me}-LDLAS	2	12.15	1716.78	1716.8 $[M+H]^+$
Q420A	F	AG-R_5^{Me}-LD-S_5^{Me}-LDLAS	3	11.1	1660.8	1660.4 $[M+H]^+$
L423A	F	QG-R_5^{Me}-AD-S_5^{Me}-LDLAS	3	10.0	1675.8	1675.3 $[M+H]^+$
D424A	F	QG-R_5^{Me}-LA-S_5^{Me}-LDLAS	3	10.9	1673.8	1673.4 $[M+H]^+$
L426A	F	QG-R_5^{Me}-LD-S_5^{Me}-ADLAS	3	9.8	1675.8	1675.3 $[M+H]^+$
D427A	F	QG-R_5^{Me}-LD-S_5^{Me}-LALAS	3	11.0	1673.8	1673.4 $[M+H]^+$
L428A	F	QG-R_5^{Me}-LD-S_5^{Me}-LDAAS	3	10.1	1675.8	1675.3 $[M+H]^+$
S430A	F	QG-R_5^{Me}-LD-S_5^{Me}-LDLAA	3	11.0	1700.8	1701.4 $[M+H]^+$
**2**	F	QG-R_5^{Me}-LD-S_5^{Me}-LDLA	2	12.55	1629.8	1629.7 $[M+H]^+$
**3**	F	QG-R_5^{Me}-LD-S_5^{Me}-LDL	2	12.16	1558.7	1558.6 $[M+H]^+$
**4**	F	QG-R_5^{Me}-LD-S_5^{Me}-LD	2	11.17	1445.6	1445.5 $[M+H]^+$
**5** (Me/Me)	Ac	G-R_5^{Me}-LD-S_5^{Me}-LDL	1	11.38	938.6	938.5 $[M+H]^+$
	F	G-R_5^{Me}-LD-S_5^{Me}-LDL	2	13.19	1430.65	1431.6 $[M+H]^+$
**6**	F	R_5^{Me}-LD-S_5^{Me}-LDL	4	17.71	1373.63	1373.5 $[M+H]^+$
**7** (H/H)	Ac	G-R_5^{H}-LD-S_5^{H}-LDL	1	9.61	910.52	910.5 $[M+H]^+$
	F	G-R_5^{H}-LD-S_5^{H}-LDL	2	11.17	1402.62	1402.5 $[M+H]^+$
**8** (H/Me)	Ac	G-R_5^{H}-LD-S_5^{Me}-LDL	1	10.13	924.54	924.5 $[M+H]^+$
	F	G-R_5^{H}-LD-S_5^{Me}-LDL	2	12.67	1416.64	1416.6 $[M+H]^+$
**9** (Me/H)	Ac	G-R_5^{Me}-LDS-S_5^{H}-LDL	1	10.17	924.54	924.5 $[M+H]^+$
	F	G-R_5^{Me}-LD-S_5^{H}-LDL	2	12.59	1416.64	1416.6 $[M+H]^+$
**10** (Me/Et)	Ac	G-R_5^{Me}-LD-S_5^{Et}-LDL	1	11.74	952.57	952.6 $[M+H]^+$
	F	G-R_5^{Me}-LD-S_5^{Et}-LDL	2	13.39	1444.67	1444.6 $[M+H]^+$
**11** (Et/Me)	Ac	G-R_5^{Et}-LD-S_5^{Me}-LDL	1	11.93	952.57	952.6 $[M+H]^+$
	F	G-R_5^{Et}-LD-S_5^{Me}-LDL	2	13.37	1444.67	723.0 $[M+2H]^{2+}$
**12** (EtEt)	Ac	G-R_5^{Et}-LD-S_5^{Et}-LDL	1	12.18	966.59	966.6 $[M+H]^+$
	F	G-R_5^{Et}-LD-S_5^{Et}-LDL	2	13.31	1458.69	1458.6 $[M+H]^+$

[a] F: FITC-peg$_2$, Ac: acetyl; [b] gradient 1: 30 % B to 70 % B in 20 min (3 min pre-run 30 % B); gradient 2: 30 % B to 95 % B in 20 min (3 min pre-run 30 % B); gradient 3: 0 % B to 70 % B in 10 min; gradient 4: 30 % B to 60 % B in 20 min [c] calculated molecular mass to charge ratio (m/z) for charged ions $[M+H]^+$ or $[M+2H]^{2+}$.

Table S2: Dissociation constants K_d of peptides considered in alanine scan of peptide **1** were determined by direct FP assay.

Peptide	Sequence	K_d / µM
**Q420A**	FITC-peg-AG-R_5^{Me}-LD-S_5^{Me}-LDLAS	0.046±0.001
**L423A**	FITC-peg-QG-R_5^{Me}-AD-S_5^{Me}-LDLAS	0.140±0.001
**D424A**	FITC-peg-QG-R_5^{Me}-LA-S_5^{Me}-LDLAS	0.154±0.001
**L426A**	FITC-peg-QG-R_5^{Me}-LD-S_5^{Me}-ADLAS	7.200±0.040
**D427A**	FITC-peg-QG-R_5^{Me}-LD-S_5^{Me}-LALAS	5.26±0.070
**L428A**	FITC-peg-QG-R_5^{Me}-LD-S_5^{Me}-LDAAS	3.690±0.020
**S430A**	FITC-peg-QG-R_5^{Me}-LD-S_5^{Me}-LDLAA	0.072±0.005

Table S3: Dissociation constants K_d of peptide **1** as well as *N*- and *C*-terminal truncated derivatives of **1** were determined by direct FP assay.

Peptide	Sequence	K_d / µM
**1**	FITC-peg-QG-R_5^{Me}-LD-S_5^{Me}-LDLAS	0.046±0.001
**2**	FITC-peg-QG-R_5^{Me}-LD-S_5^{Me}-LDLA	0.140±0.001
**3**	FITC-peg-QG-R_5^{Me}-LD-S_5^{Me}-LDL	0.154±0.001
**4**	FITC-peg-QG-R_5^{Me}-LD-S_5^{Me}-LD	7.200±0.040
**5** (Me/Me)	FITC-peg-G-R_5^{Me}-LD-S_5^{Me}-LDL	5.26±0.070
**6**	FITC-peg-R_5^{Me}-LD-S_5^{Me}-LDL	3.690±0.020

Table S4: Dissociation constants K_d, half maximal inhibitory concentrations IC_{50} and inhibitory constants K_i of **5** (Me/Me) derivatives with varying substitution pattern at X_R3 and X_S6 were determined by direct FP and FP competition assay, respectively.

Peptide	Sequence[a]	K_d / µM	IC_{50} / µM	K_i / µM
**1**	QG-R_5^{Me}-LD-S_5^{Me}-LDLAS	0.046±0.020	6.335±0.625	1.893±1.965
**7** (H/H)	G-R_5^{H}-LD-S_5^{H}-LDL	>200	>100	>10
**8** (H/Me)	G-R_5^{H}-LD-S_5^{Me}-LDL	~50	>100	>10
**9** (Me/H)	G-R_5^{Me}-LD-S_5^{H}-LDL	~50	>100	>10
**5** (Me/Me)	G-R_5^{Me}-LD-S_5^{Me}-LDL	1.750±0.086	9.689±1.298	3.187±2.029
**10** (Me/Et)	G-R_5^{Me}-LD-S_5^{Et}-LDL	1.520±0.169	9.872±2.156	3.258±2.136
**11** (Et/Me)	G-R_5^{Et}-LD-S_5^{Me}-LDL	0.560±0.047	3.032±0.159	0.614±1.944
**12** (Et/Et)	G-R_5^{Et}-LD-S_5^{Et}-LDL	0.055±0.046	8.622±0.505	2.775±1.970

[a] with N-terminal FITC-peg$_2$ modification for the determination of K_d-values by direct FP assays, *N*-terminal acetyl modification for the determination of IC_{50}-values by FP competition assays.

Table S5: Effect of restraints on the ligand flexibility in complex and solvent legs of FEP simulations. Average RMSD-values of ligands to the corresponding trajectory centroid structures. Trajectories were overlaid by ligand backbone and the crosslink heavy atoms.

	None (0 kcal·mol^{-1}·Å^{-2})	Soft (0.1 kcal·mol^{-1}·Å^{-2})	Medium (1 kcal·mol^{-1}·Å^{-2})	Hard (10 kcal·mol^{-1}·Å^{-2})
free	0.933 Å	0.718 Å	0.554 Å	0.389 Å
complex	0.758 Å	0.666 Å	0.503 Å	0.345 Å

Table S6: ΔpK_d-values for **5** (Me/Me) derivatives relative to **5** (Me/Me) calculated by FEP applying various restraining force constants (none: 0; soft: 0.1, middle: 1, hard: 10 kcal·mol^{-1}·Å^{-2}) and experimentally determined ΔpK_d-values for **5** (Me/Me) derivatives relative to **5** (Me/Me) by FP assays.

Peptide	None	Soft	Middle	Hard	Experimentally determined
**7** (H/H)	-1.75±0.37	-2.16±0.81	-2.31±0.38	-2.15±0.30	< -3
**8** (Me/H)	-1.19±0.38	-1.62±0.61	-2.11±0.45	-1.87±0.46	-1.57±0.03
**5** (Me/Me)	0±0.29	0±0.29	0±0.29	0±0.29	0±0.02
**11** (Et/Me)	-0.05±0.35	-0.02±0.66	0.46±0.33	0.28±0.46	0.495±0.04
**12** (Et/Et)	0.57±0.32	0.38±0.87	0.43±0.34	0.63±0.43	0.508±0.04

Table S7: Calculated Δp$K_d^{*,c}$ -values for **7** (H/H), **8** (Me/H), **5** (Me/Me), **11** (Et/Me) and **12** (Et/Et) relative to **5** (Me/Me) derived from REST MD calculations as described in section 1.8. Errors determined by block averaging and account for 1σ.

Peptide	Δp$K_d^{*,c}$
**7** (H/H)	-0.83±0.27
**8** (Me/H)	-0.29±0.32
**5** (Me/Me)	0
**11** (Et/Me)	0.14±0.22
**12** (Et/Et)	0.04±0.37

Table S8: Reference compounds used to establish a calibration curve for LogD determination. Retention times t_R were obtained applying the linear gradient 0 min / 0 % B, 20 min / 95 % B, 30 min / 95 % B, 30.1 min / 0 % B, 34.1 min / 0 % B with an aqueous 50 mM ammonium acetate solution (pH 7.4) as A and acetonitrile as B, corresponding LogD-values are received from literature.

Reference compound	t_R / min	LogD
Sulpiride	9.17±0.021	-1.15
Metoprolol	10.21±0.024	-0.06
Labetolol	12.14±0.010	1.07
Diltiazem	15.31±0.002	2.70
Triphenylene	19.92±0.001	5.49

Table S9: Determined LogD-values. Retention times t_R were obtained applying the linear gradient 0 min / 0 % B, 20 min / 95 % B, 30 min / 95 % B, 30.1 min / 0 % B, 34.1 min / 0 % B with an aqueous 50 mM ammonium acetate solution (pH 7.4) as A and acetonitrile as B., corresponding LogD-values were calculated based on equation (7).

Peptide	t_R / min	LogD
**1**	12.58±0.197	1.15±0.068
**7** (H/H)	11.70±0.007	0.63±0.019
**8** (H/Me)	12.37±0.003	1.03±0.031
**9** (Me/H)	12.35±0.003	1.01±0.031
**5** (Me/Me)	12.75±0.003	1.26±0.038
**10** (Me/Et)	12.78±0.009	1.28±0.040
**11** (Et/Me)	12.88±0.003	1.33±0.041
**12** (Et/Et)	12.92±0.001	1.35±0.041

Table S10: Calculated dynamic non-polar and polar surface area (NPSA and PSA, respectively) of peptide **7** (H/H), **9** (H/Me), **5** (Me/Me), **11** (Et/Me) and **12** (Et/Et).

Peptide	NPSA / nm^2	PSA / nm^2
**7** (H/H)	7.630±0.027	4.333±0.017
**9** (H/Me)	7.837±0.034	4.043±0.033
**5** (Me/Me)	7.678±0.025	3.663±0.038
**11** (Et/Me)	7.836±0.048	3.575±0.034
**12** (Et/Et)	7.952±0.056	3.560±0.021

Table S11: Data collection and refinement statistics for 14-3-3ζΔC/**11** (Et/Me)-complex (3.7 Å resolution, PDB ID 6rlz). Values in parenthesis refer to the highest resolution shell.

	**14-3-3ζΔC/11(Et/Me)**
Data collection	
Space group	$P6_4$
Cell dimensions	
a, b, c (Å)	94.8, 98.8, 98.8
α, β, γ (°)	90, 90, 120
Resolution (Å)	50.0-3.7 (3.8-3.7)
R_{meas} (%)	17.7 (100)
$CC_{1/2}$	99.2 (92.1)
I / σ	16.02 (4.91)
Completeness (%)	99.1 (97.5)
Redundancy	33.0 (32.5)
Refinement	
Resolution (Å)	43.81-3.7 (3.8-3.7)
Total no. reflections	186022 (14000)
Unique no. reflections	5069 (431)
R_{work} / R_{free}	0.216/0.292
No. total atoms	3503
protein	A: 1740, B: 1615
ligand/ion	C: 63, D: 57
waters	28
Mean B-factor	120.23
R.m.s.deviations	
bond lenghts (Å)	0.002
bond angles (°)	0.841
Ramachandran (%)	
preferred regions	92.95
allowed regions	6.83
not allowed regions	0.22

3. Supporting Figures

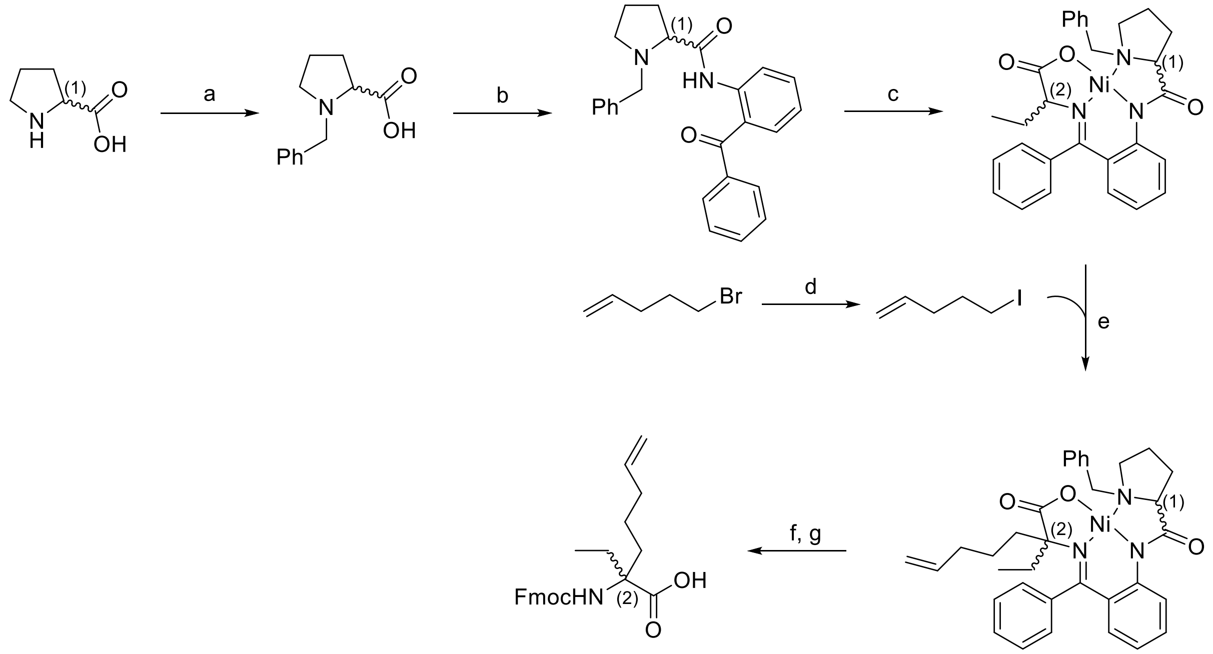

Scheme S1: Synthesis of α-ethyl-α-alkenyl amino acids. (a) 3.8 eq KOH, 2.5 eq BnBr, *i*PrOH, 40 °C, 6 h; (b) 2.2 eq 1-methylimidazole, 1 eq methanesulfonyl chloride, 0.9 eq *o*-aminobenzophenone, DCM, 50 °C, overnight; (c) 2 eq nickel(II) nitrate hexahydrate, 2 eq *rac*-homoalanine, 8 eq KOH, MeOH, 80 °C, 2 h; (d) 2 eq/4 eq, NaI, acetone, 60 °C, 2 h; (e) 1.5 eq alkenyl iodide, 10 eq KOH, DMF, room temperature, 6 h; (f) conc. HCl, MeOH, 80 °C, 1 h; (g) Na_2CO_3, 1.5 eq Fmoc-OSu, H_2O/dioxane, room temperature, 7 d.

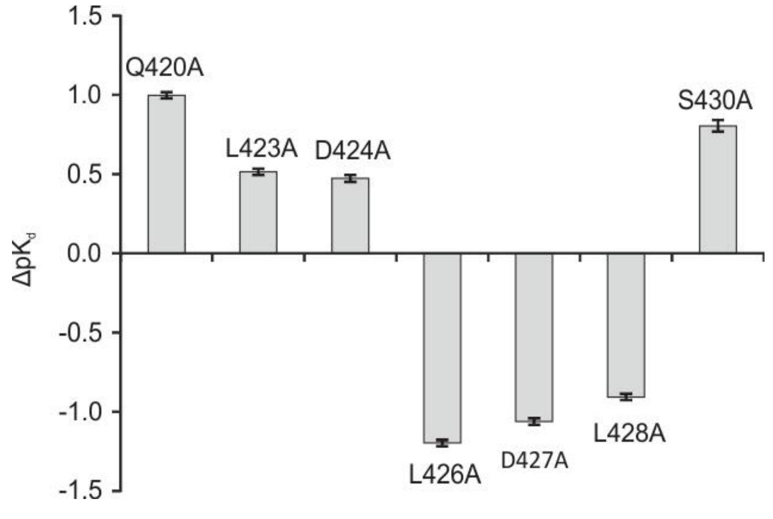

Figure S1: Differences between pK_d-values of peptides considered in alanine scan and peptide **1**. FP assays were performed in triplicates (errors account for 1 σ).

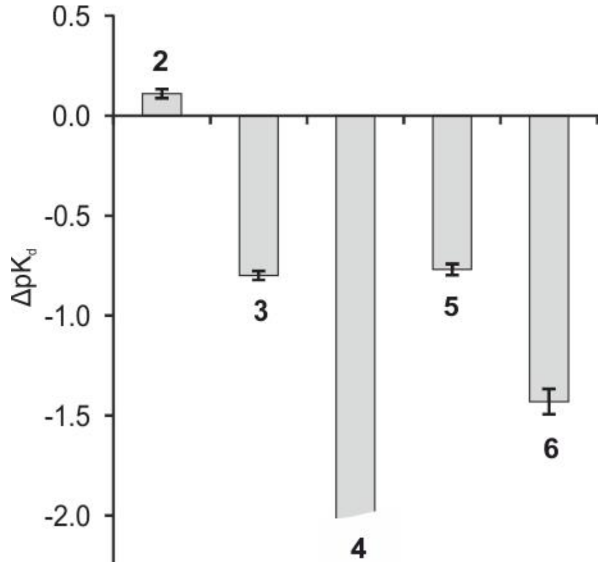

Figure S2: Differences between pK_d-values of truncated peptides (**2** – **6**) and peptide **1**. FP assays were performed in triplicates (errors account for 1 σ).

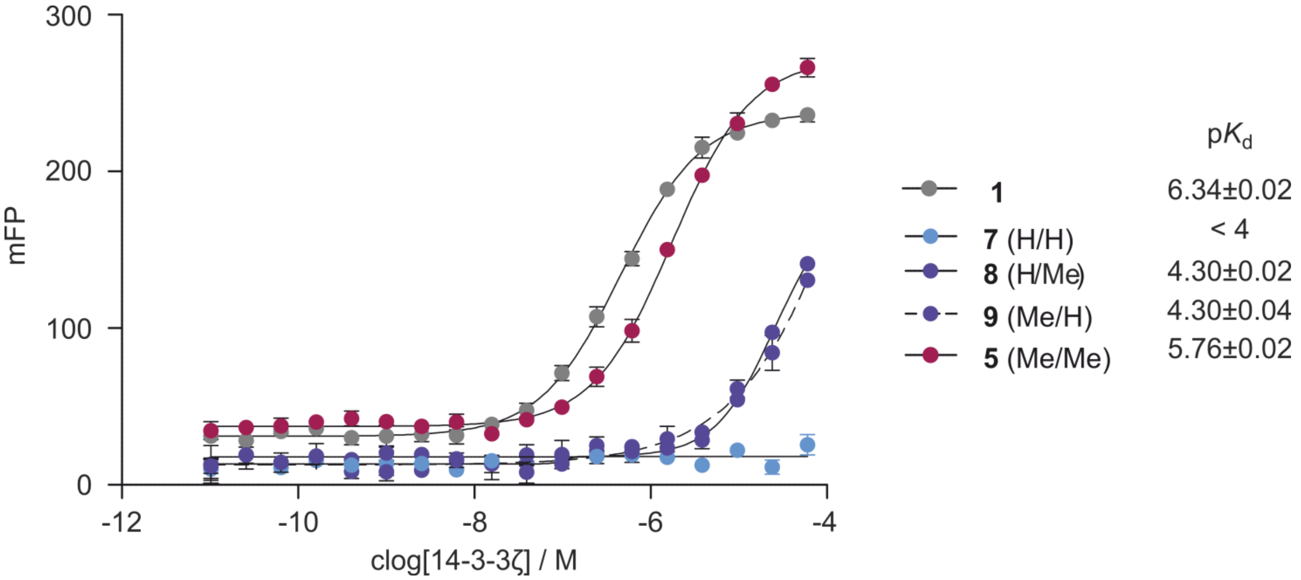

Figure S3: FP binding curves of H-modified derivatives (**7** – **9**) of peptide **5** (Me/Me) including peptide **1** and **5** with corresponding pK_d-values measured in FP assays (triplicates, errors account for 1 σ).

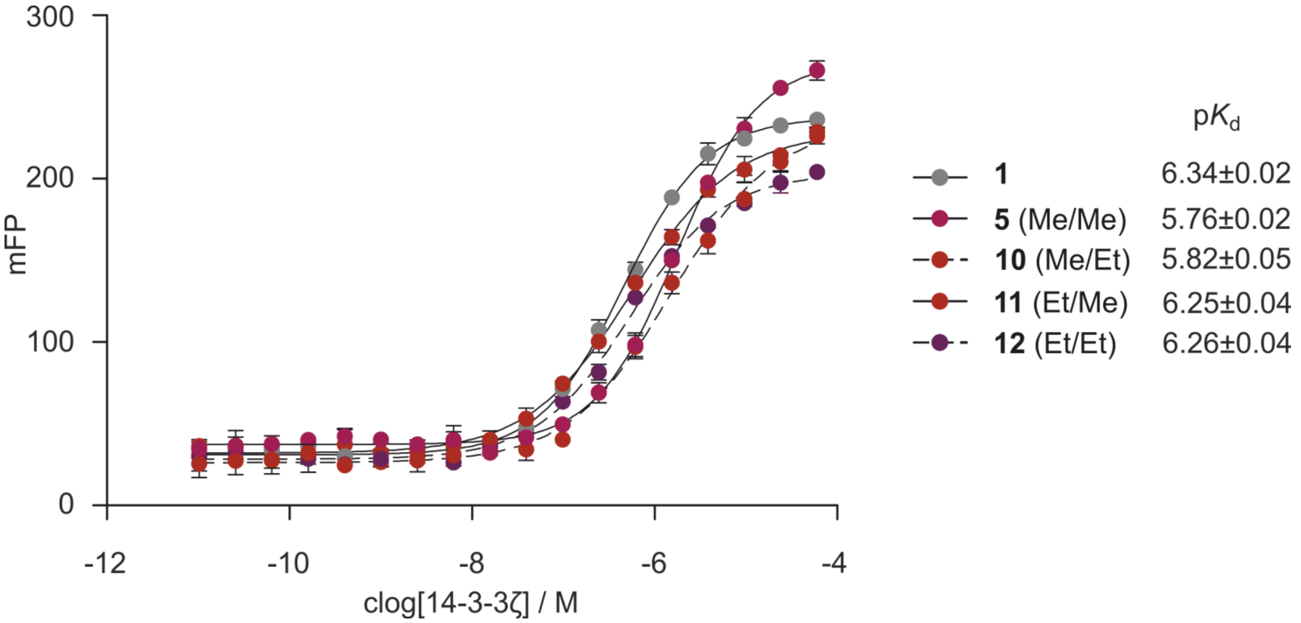

Figure S4: FP binding curves of Et-modified derivatives (**10** – **12**) of peptide **5** (Me/Me) including peptide **1** and **5** with corresponding pK_d-values measured in FP assays (triplicates, errors account for 1 σ).

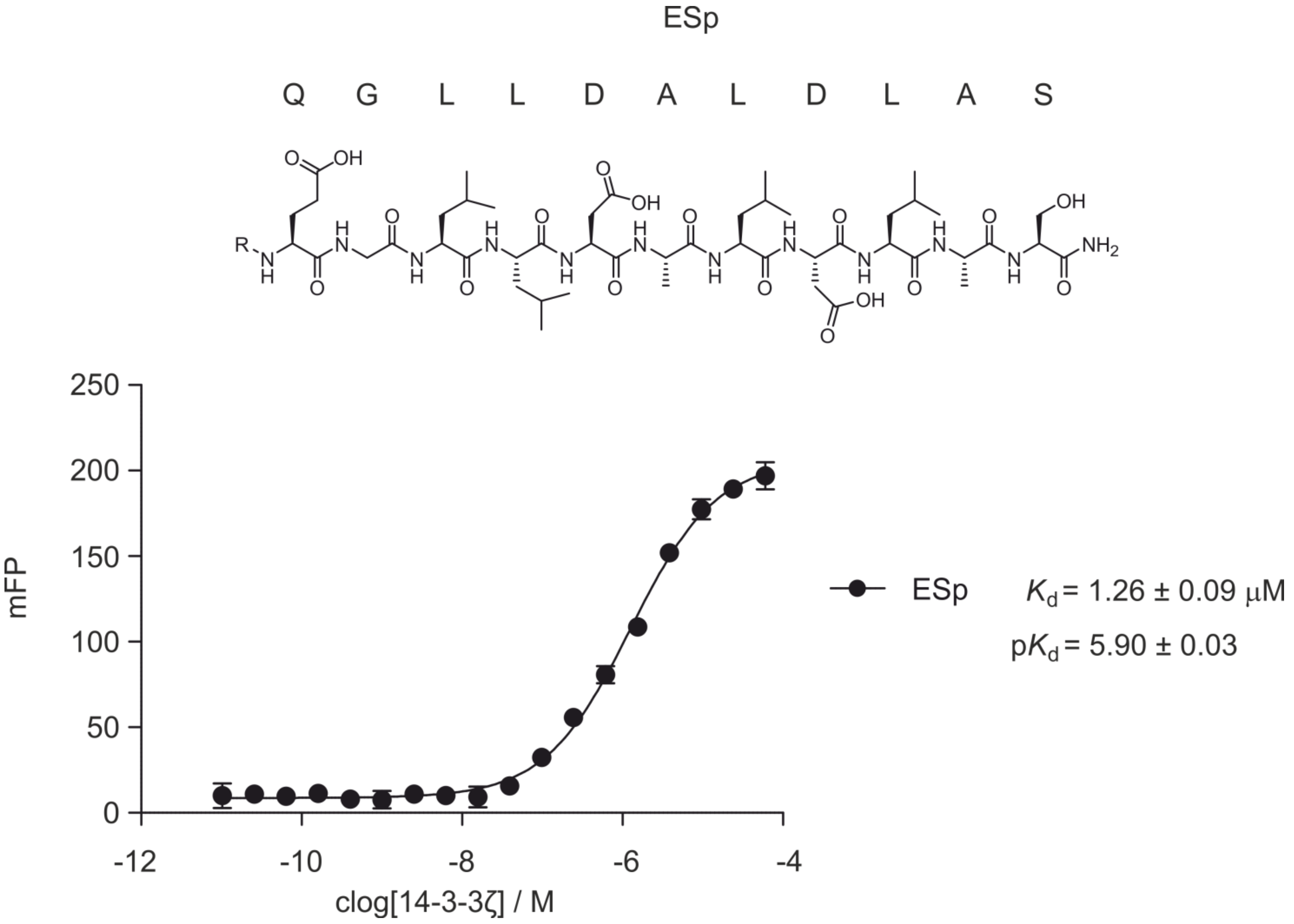

Figure S5: FP binding curve of 14-3-3 binding sequence of exoenzmye S (H_2N-QGLLDLALDLAS-$CONH_2$, ESp) with R = FITC-peg$_2$ including corresponding K_d- and pK_d-values measured in FP assays (triplicates, errors account for 1 σ).

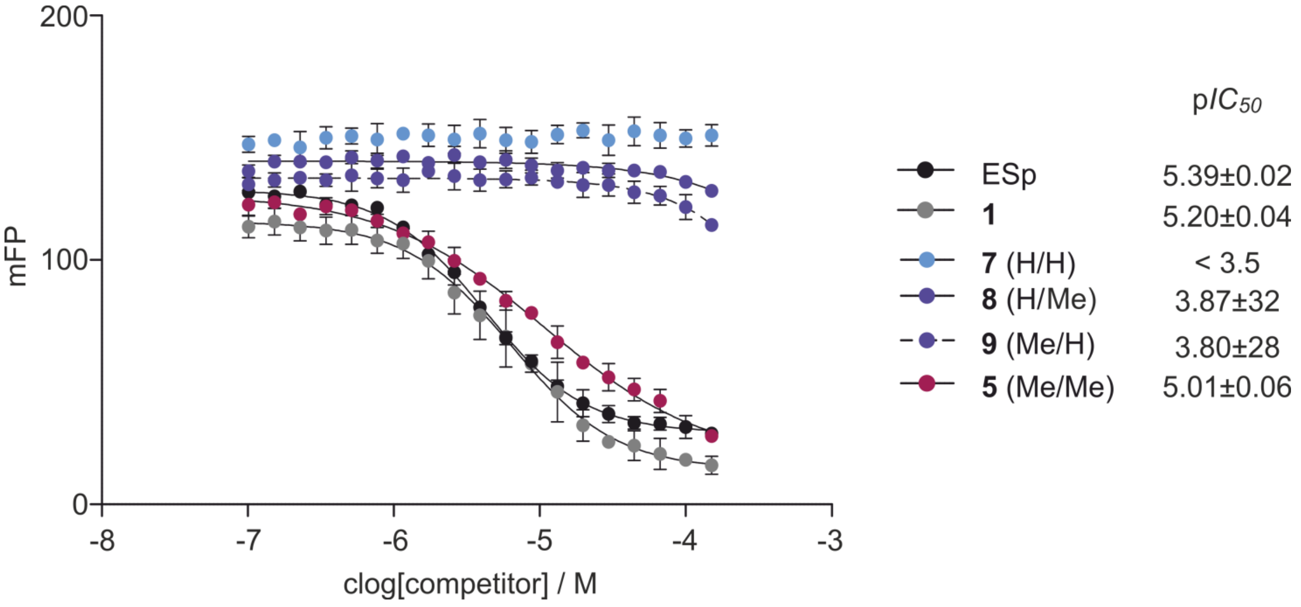

Figure S6: FP-based displacement assay of *N*-terminally acetylated, H-modified derivatives (**7** – **9**) of peptide **5** (Me/Me) as well as peptide **1** and **5** replacing *N*-terminally FITC-peg-labeled ESp from the 14-3-3 binding groove with corresponding pIC_{50}-values measured in triplicates (errors account for 1 σ).

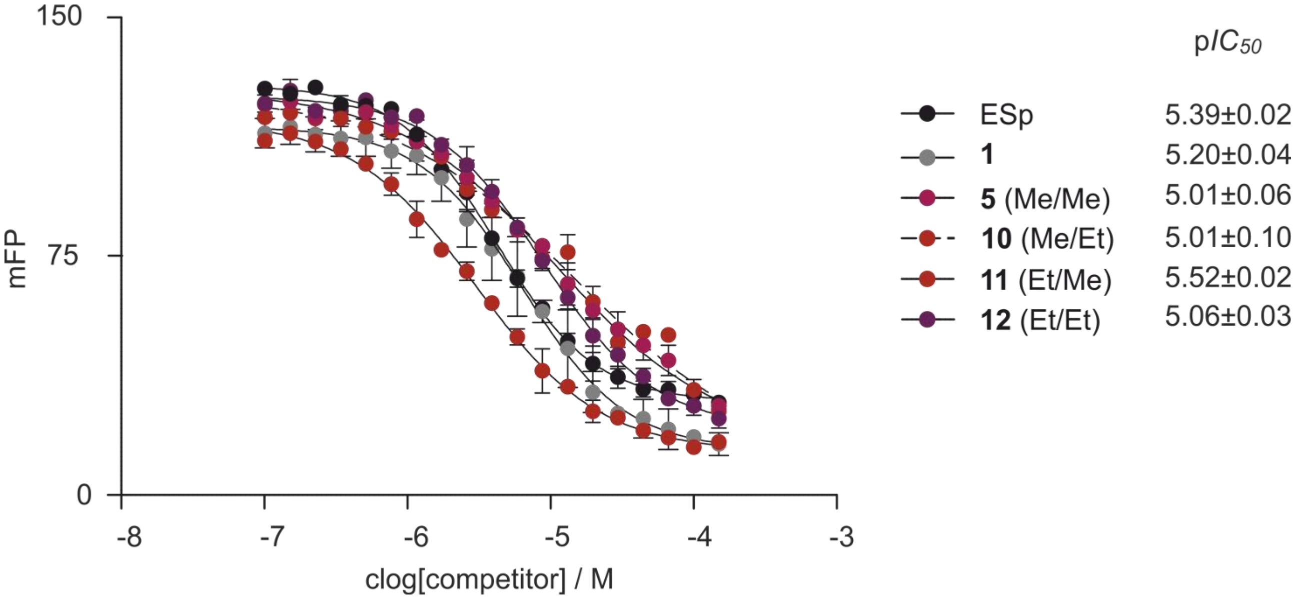

Figure S7: FP-based displacement assay of *N*-terminally acetylated, Et-modified derivatives (**10** – **12**) of peptide **5** (Me/Me) as well as peptide **1** and **5** replacing *N*-terminally FITC-peg-labeled ESp from the 14-3-3 binding groove with corresponding pIC_{50}-values measured in triplicates (errors account for 1 σ).

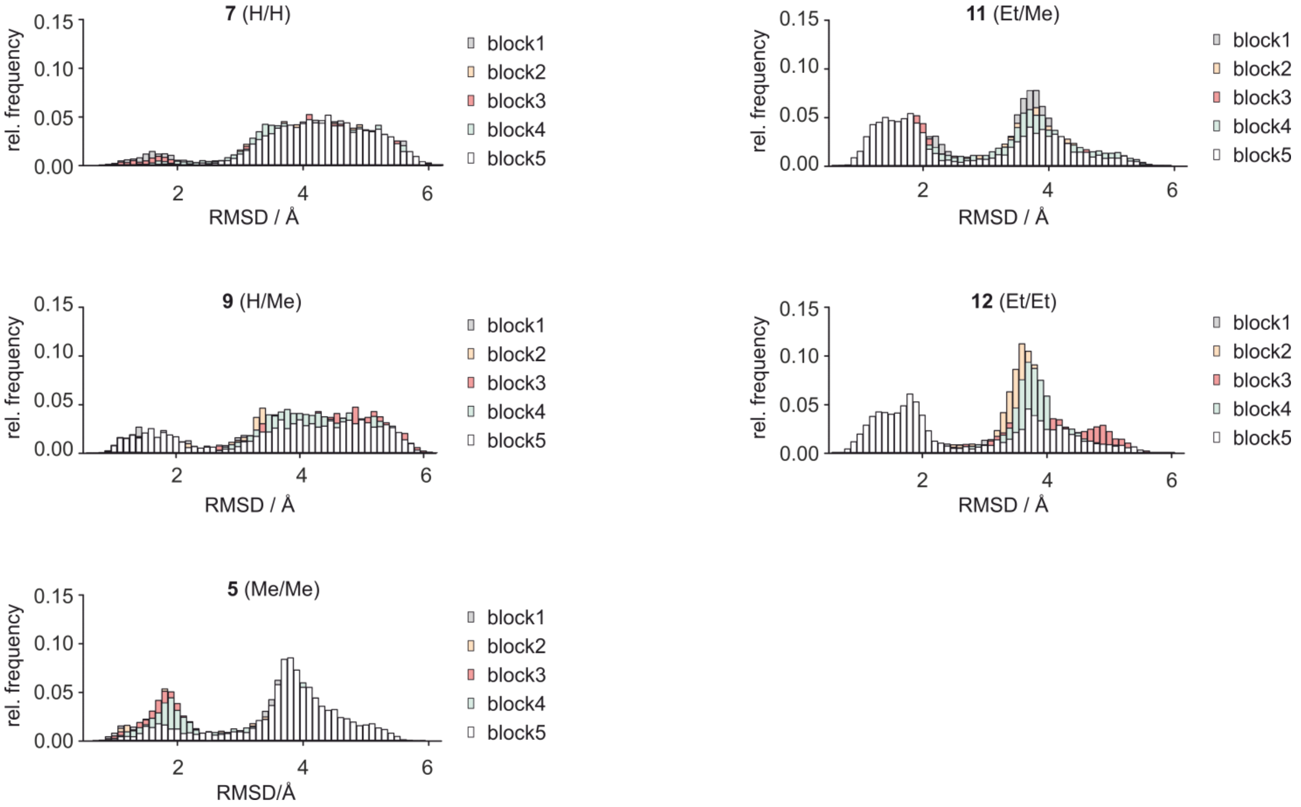

Figure S8: Ligand RMSD distributions in 2.5 µs REST MD simulations with 8 replicas for selected peptides with varying substitution pattern (**7**, H/H; **9**, Me/H; **5**, Me/Me; **11**, Et/Me; **12**, Et/Et). The 2.5 µs simulations were then split into five blocks (block1 - block5) of 0.5 µs each.

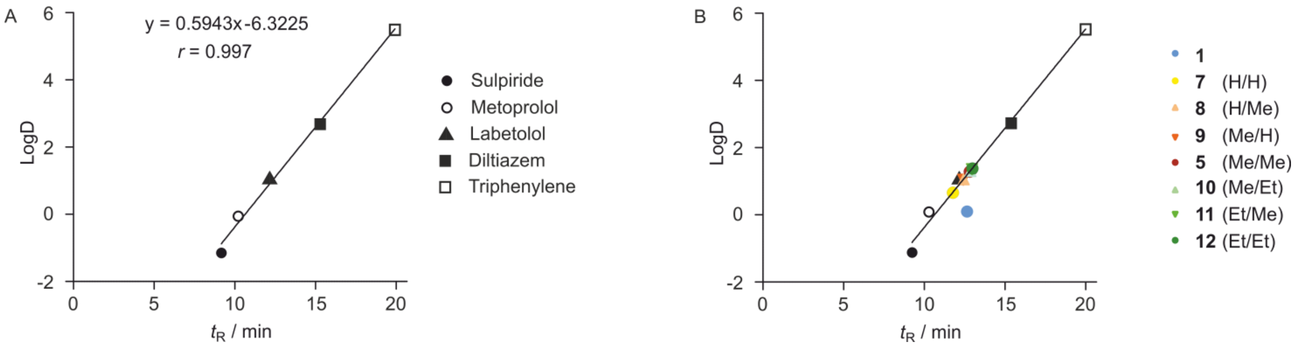

Figure S9: A) Calibration curve for LogD determination based on reference compounds with known LogD-values (Pearson correlation coefficient r = 0.997). Measured retention times t_R (gradient: 0 min / 0 % B, 20 min / 95 % B, 30 min / 95 % B, 30.1 min / 0 % B, 34.1 min / 0 % B with an aqueous 50 mM ammonium acetate solution (pH 7.4) as A and acetonitrile as B are plotted against the corresponding LogD-value; B) Retention times t_R of peptides obtained by applying the linear gradient 0 min / 0 % B, 20 min / 95 % B, 30 min / 95 % B, 30.1 min / 0 % B, 34.1 min / 0 % B with an aqueous 50 mM ammonium acetate solution (pH 7.4) as A and acetonitrile as B.

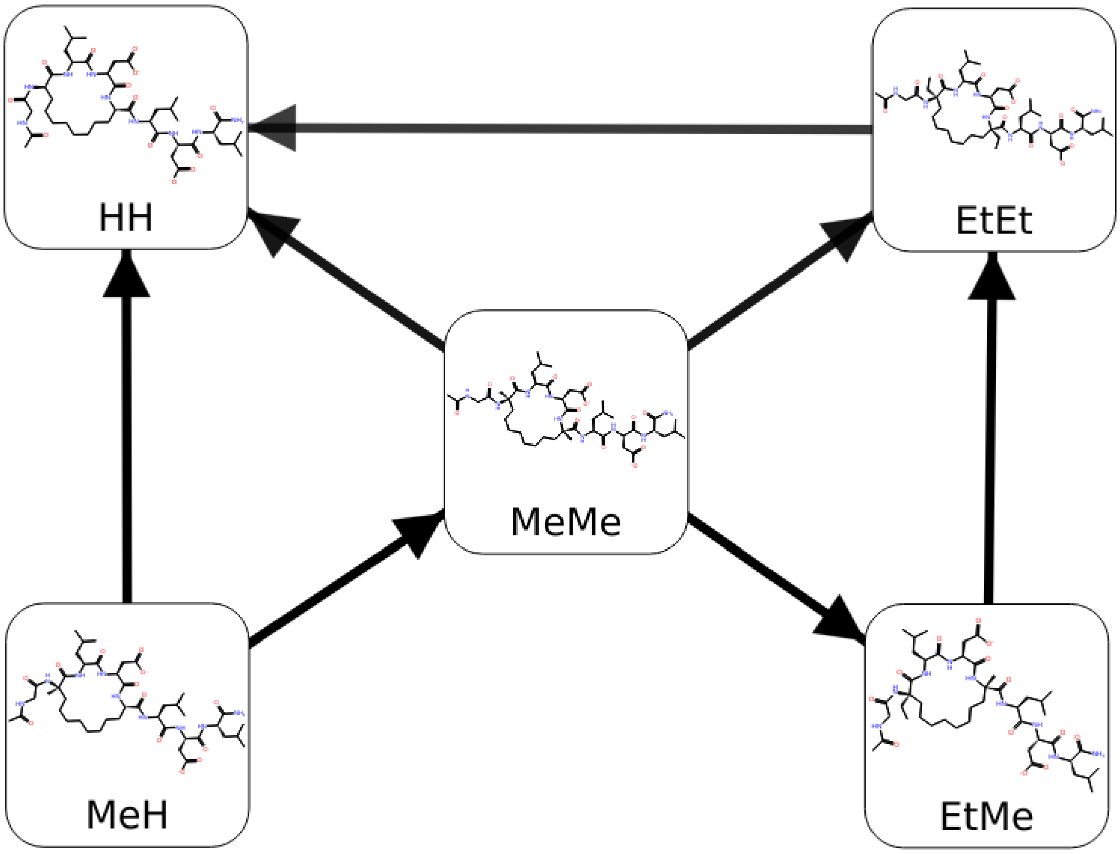

Figure S10: Graphical illustration of the ligand connections map used in FEP simulations. Nodes represent studied ligands. Arrows illustrate the edges of FEP simulation. Each edge includes two independent calculation of ΔΔG upon alchemical mutation of one ligand into the other in complex and free in solution, complex and solvent legs, correspondingly.

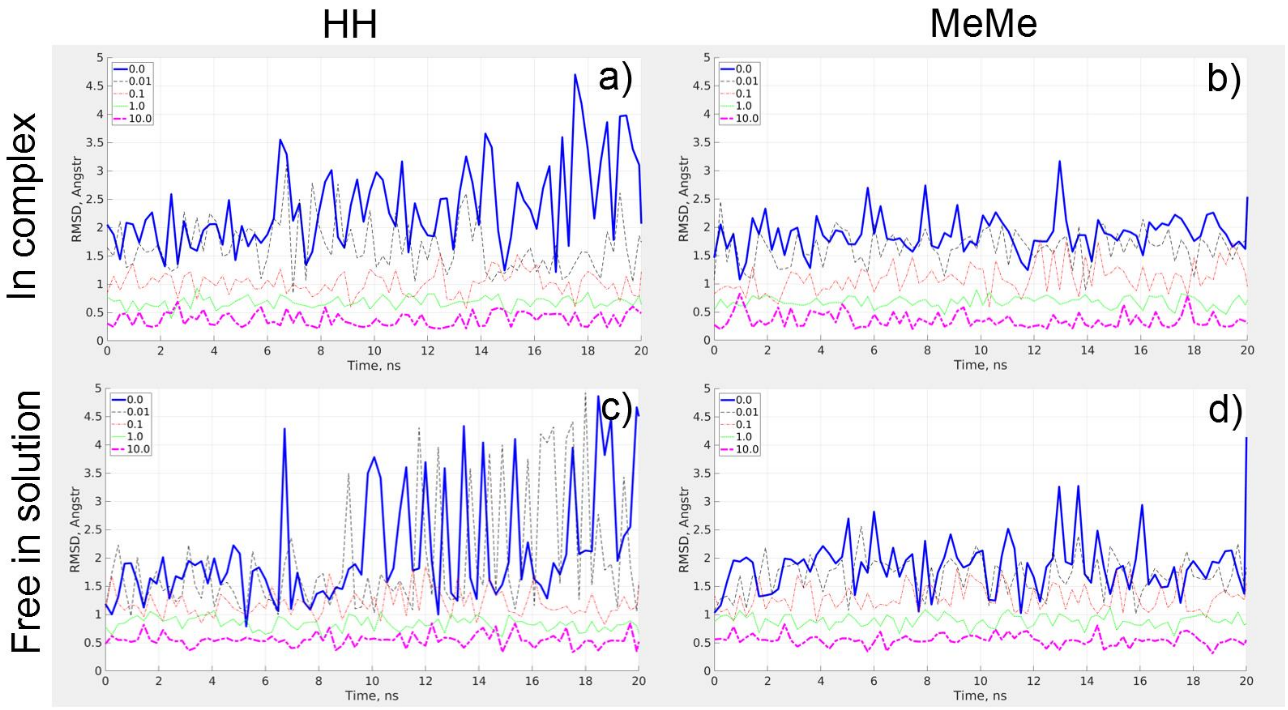

Figure S11: RMSD of ligands with respect to the reference X-ray starting point (PDB ID 4n7y) along simulation trajectories of a representative FEP edge at different position restraints force constants in kcal·mol^{-1}·Å^{-2}. First row: trajectory of protein/ligand-complex overlaid by protein backbone. Second row: trajectory of free ligand in water overlaid by ligand heavy atoms. First column: data for **7** (H/H) ligand; second column: data for **5** (Me/Me) ligand. Mind that data for the force constant of 0.01 kcal·mol^{-1}·Å^{-2} is not included in the main article for brevity. One can see that **7** (H/H) ligand start to explore wider conformational space as unbiased (no restraint force constant applied) FEP simulations evolve over time. Clearly, at least in the case of **7** (H/H) ligand, the unbiased FEP simulations are not converged in exploring relevant configuration space. Fast changes in RMSD (spikes on the RMSD curve) should be related to the stochastic replica exchange protocol implemented in FEP simulations. In the case of position restrained FEP simulations, RMSD plots are uniform over the simulation trajectory indicating that ligand explores similar configuration space over the simulation trajectory and one can expect sufficient convergence of FEP free energy calculations.

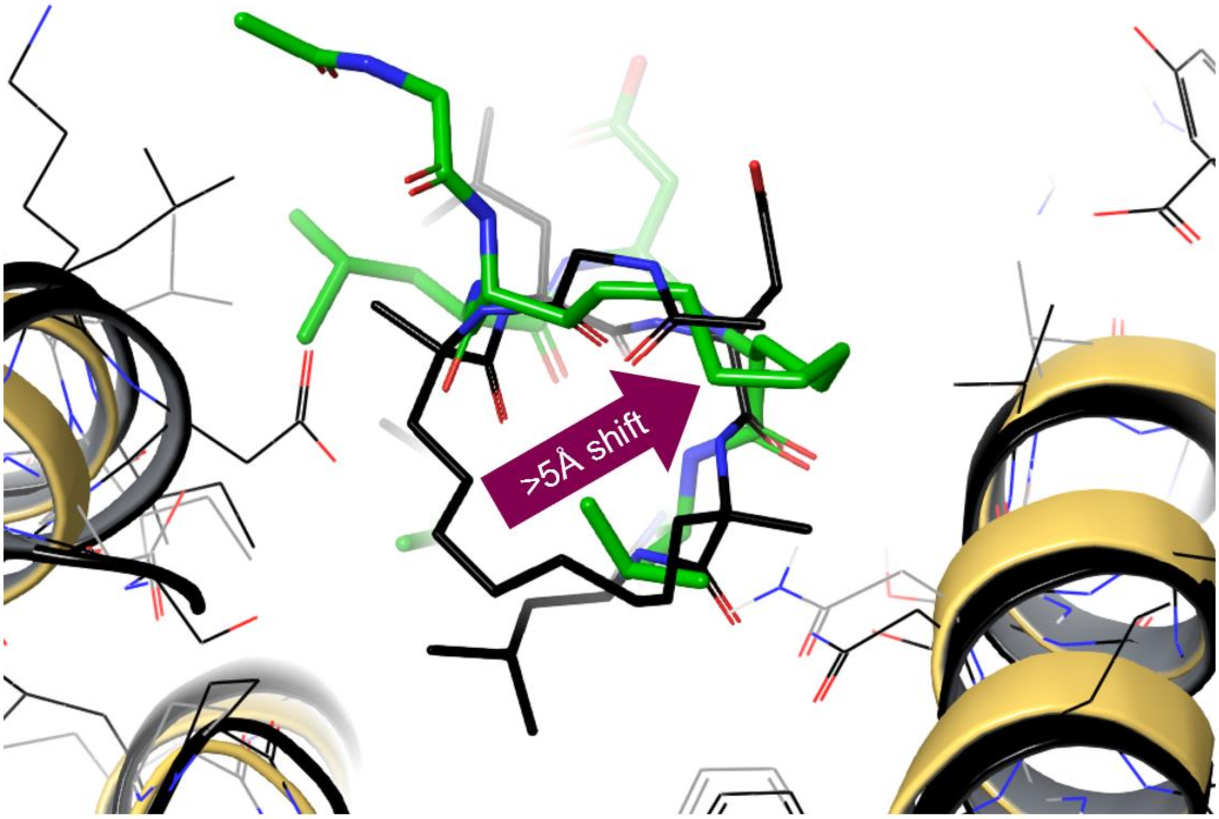

Figure S12: A representative structure from FEP trajectory of **7** (H/H) with high RMSD (green ligand, grey/yellow protein) with respect to the reference X-ray structure (black ligand and protein, PDB ID 4n7y). The error illustrates a large move of the hydrocarbon crosslink from the protein pocket into solvent. We interpret this as initiation of ligand unbinding during FEP simulations due to low affinity of **7** (H/H).

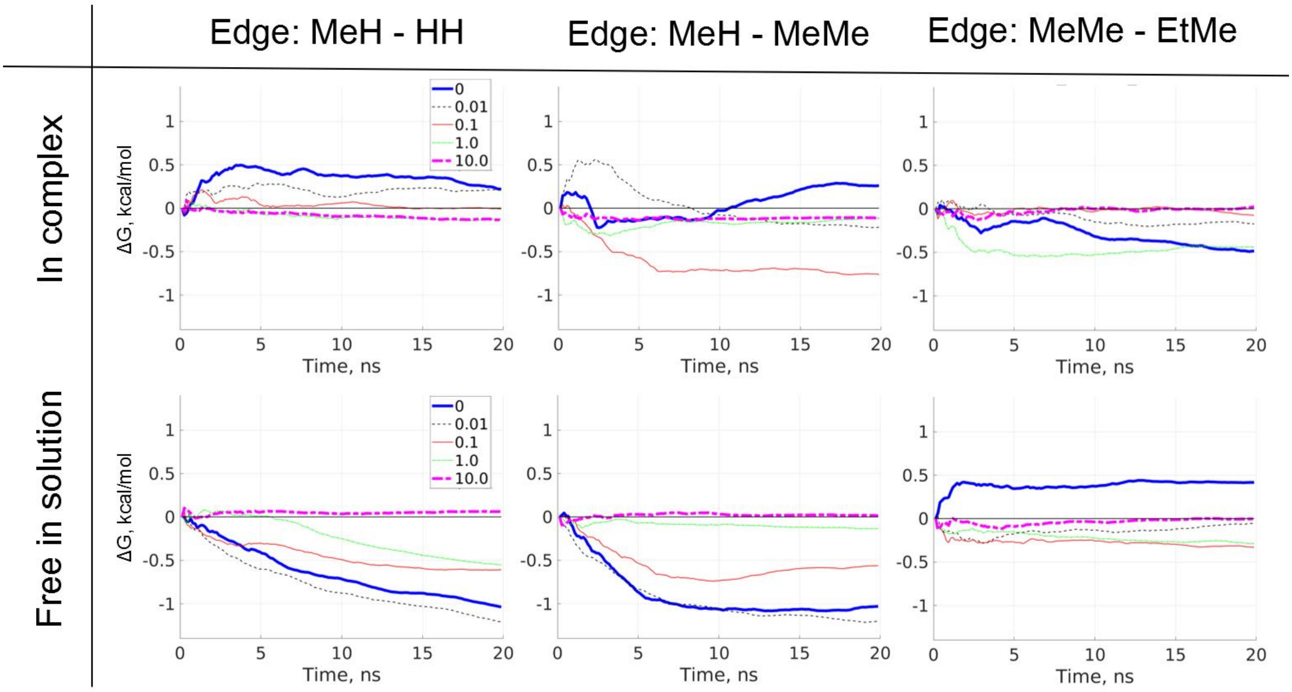

Figure S13: Running free energy estimate as a function of simulation time of some representative FEP edges (columns) for complex and solvent legs of FEP simulations (rows) at different position restraints force constants in kcal·mol^{-1}·Å^{-2}. Data for the force constant of 0.01 kcal·mol^{-1}·Å^{-2} is not included in the main article for brevity. In the case of unbiased FEP calculations (no restraint force constant applied) running free energy estimate often does not reach a plateau, indicating insufficient convergence. Particularly, for **9** (Me/H) – **7** (H/H) edge in solvent leg the drift of ΔG is high (> 0.5 kcal·mol^{-1}) even after 5 ns. Overall, the ΔG drift becomes less pronounced in the case of restrained FEP simulations with the increase of force constant. In the case of the highest studied force constant of 10 kcal·mol^{-1}·Å^{-2}, the drift becomes negligible and shows that the free energy estimates are statistically converged and reliable.

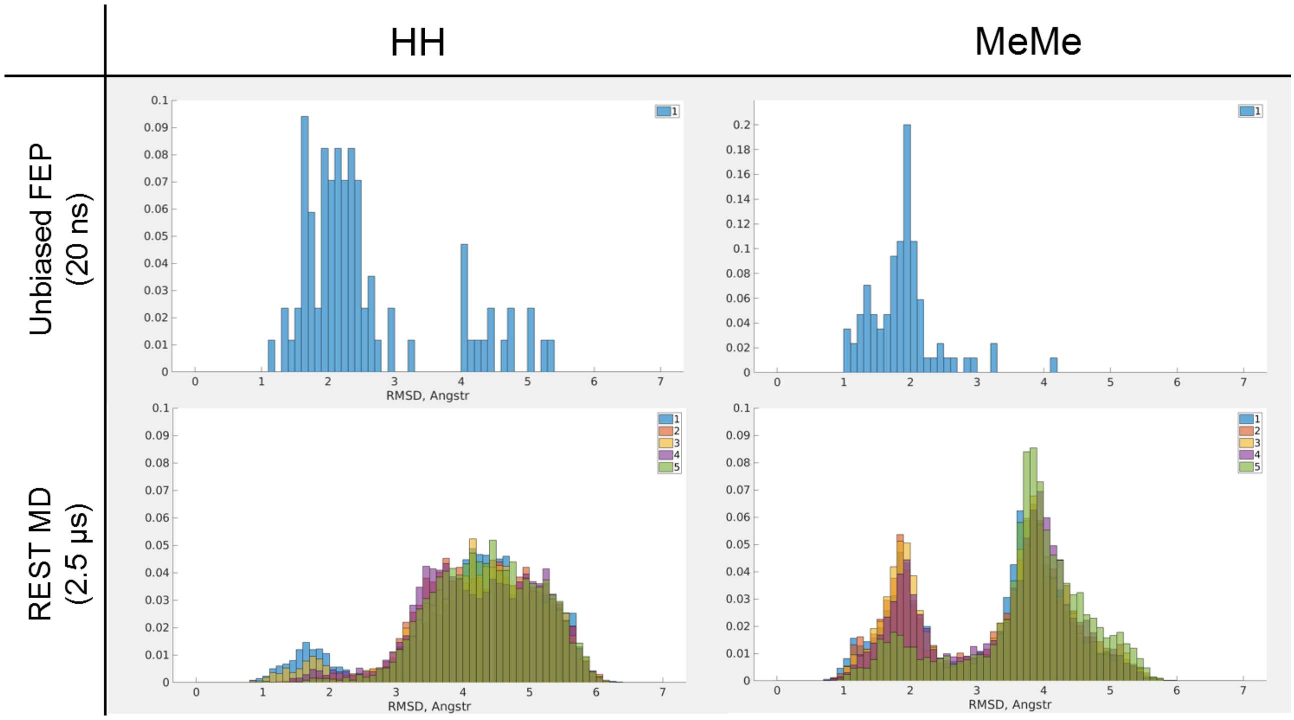

Figure S14: Distribution of ligand RMSD with respect to reference X-ray (PDB ID 4n7y) in representative unbiased FEP trajectories (first row) and in 2.5 µs REST MD simulation (second row) of **7** (H/H, first column) and **5** (Me/Me, second column) ligands in bulk water. There is a drastic difference in distributions between short unbiased FEP simulations and long REST MD simulations. This shows that the ligand does not explore a broad conformational space in FEP and predominantly stays close to initial bioactive conformation. However, in the case of REST MD simulations the bioactive conformation is not dominant.

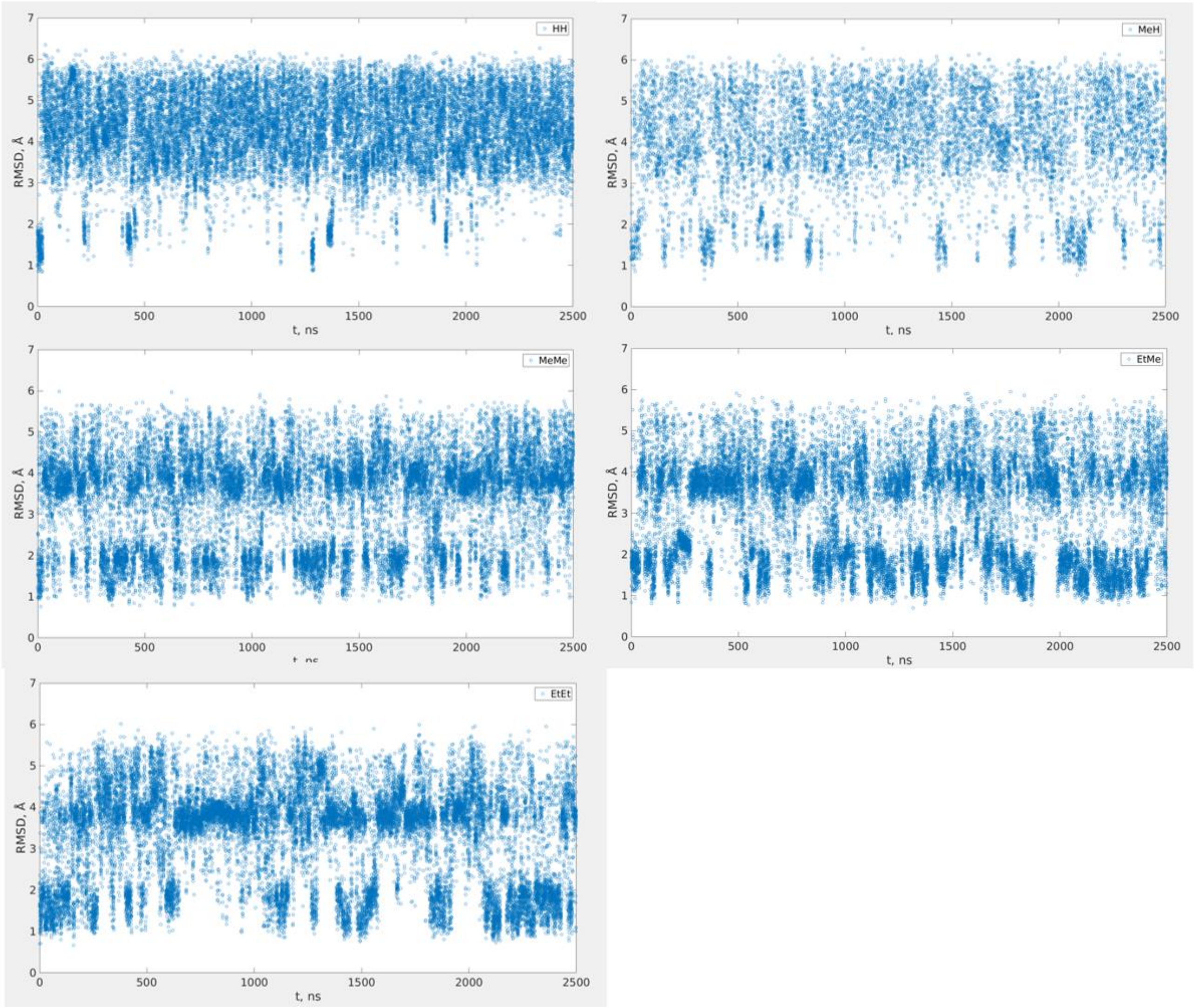

Figure S15: Ligand RMSD versus simulation time for REST MD simulation at room temperature. One can see a uniform sampling of several conformational states through the entire span of ligand RMSDs from ~1 to 6 Å. This indicates that similar conformational space is sampled along the simulation time and ligands are not trapped in local minima.

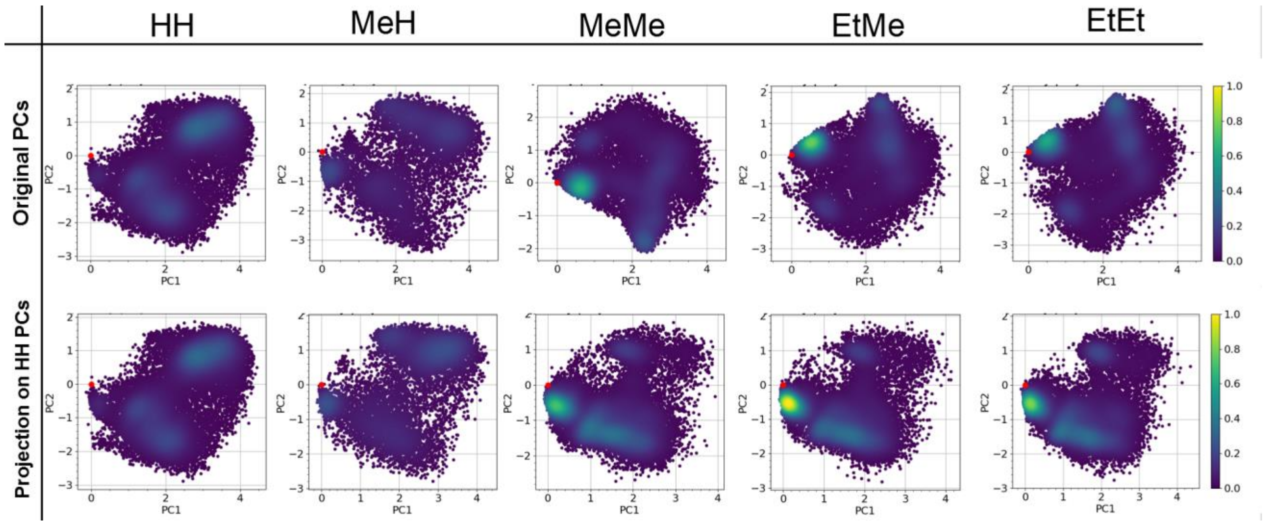

Figure S16: REST MD room temperature simulation trajectories projected on the first two principal component (PC) vectors calculated in ligand heavy atom cartesian coordinate space. First row: principal component analysis for each ligand trajectory. Second row: projection of the ligand trajectories on PC vectors derived from HH ligand trajectory. Dots are coloured proportional to the probability density in the first two PC space. The red dot is the projection of the reference X-ray conformation. One can see that similar conformational space was accessible to all ligands in REST MD simulations. There are at least 3 distinct peaks on the 2D distributions indicating various preferred conformational states of the ligands. The peak close to the reference X-ray conformation corresponds to bioactive conformation. The first two PCs account for about 60% of variation in the case of all ligands.

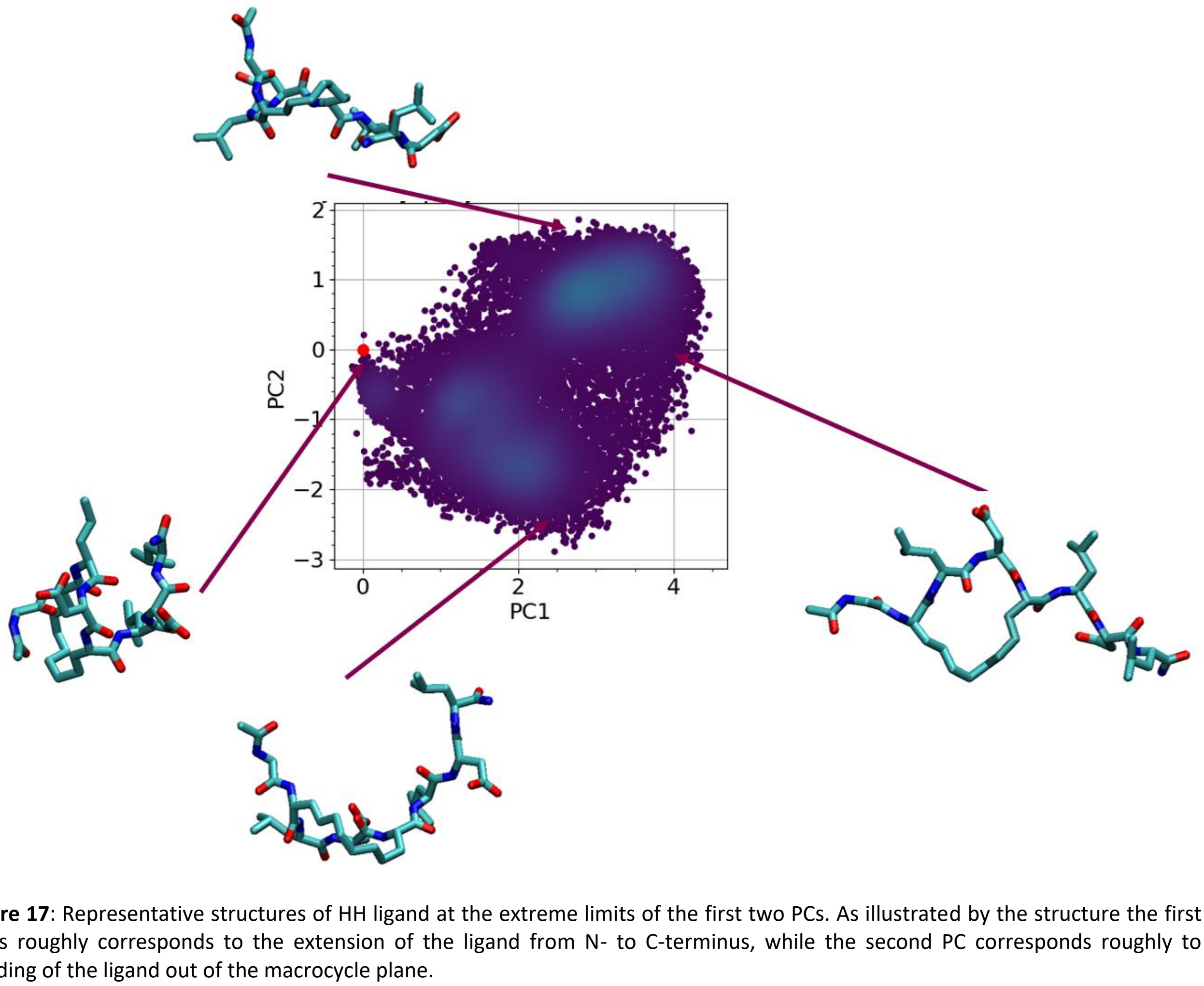

Figure 17: Representative structures of HH ligand at the extreme limits of the first two PCs. As illustrated by the structure the first PC is roughly corresponds to the extension of the ligand from N- to C-terminus, while the second PC corresponds roughly to bending of the ligand out of the macrocycle plane.

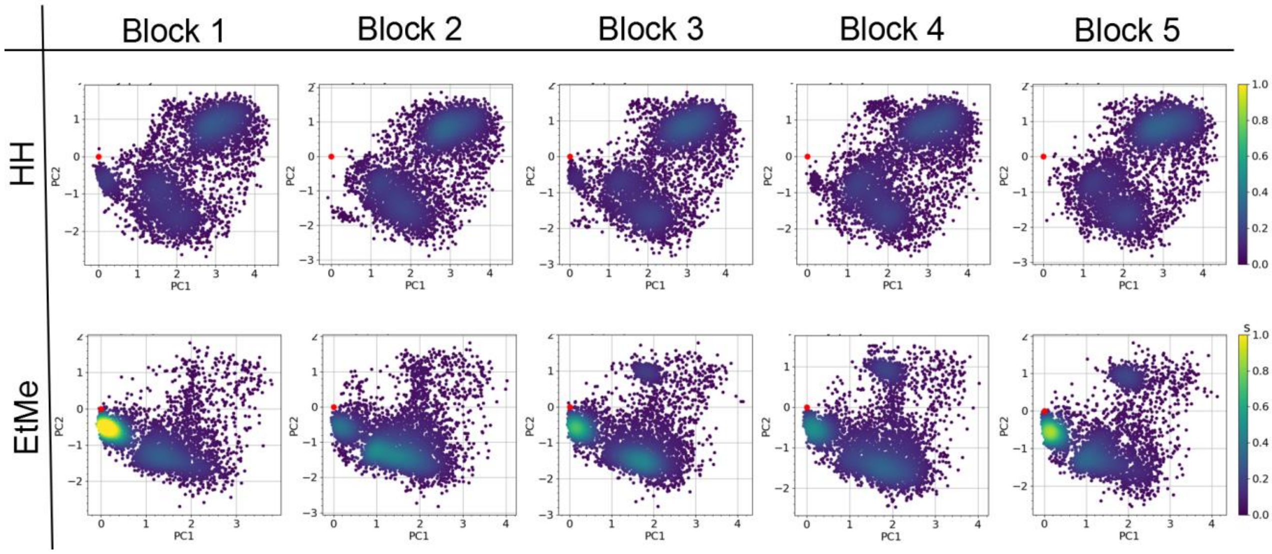

Figure S18: Block analysis of the REST MD room temperature simulation trajectories projected on the first two PC vectors derived from HH ligand trajectory. Each block represents 500 ns of the simulated trajectory. Importantly, similar space is accessible to ligands in each block of the trajectory. The variance of the peak heights allows to judge statistical uncertainly of different conformer populations.

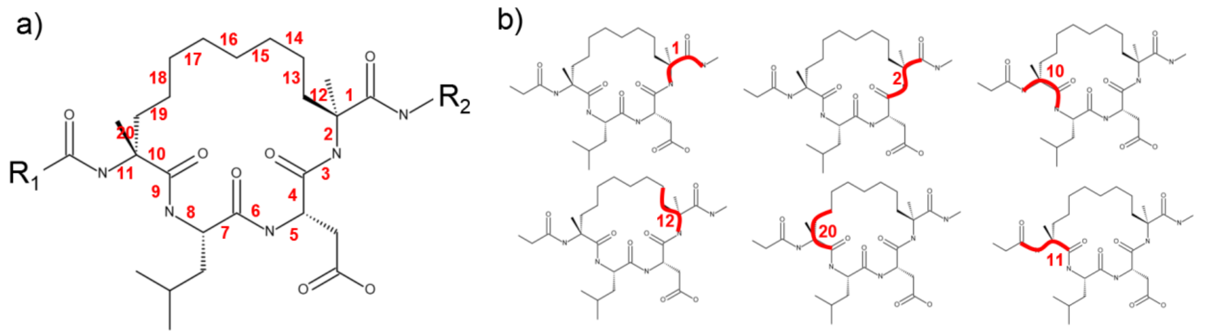

Figure S19: a) Indices of the torsion angles of the macrocycle. b) We chose 4 heavy atoms to define the dihedral angle. Unambiguous cases are explicitly marked by red line.

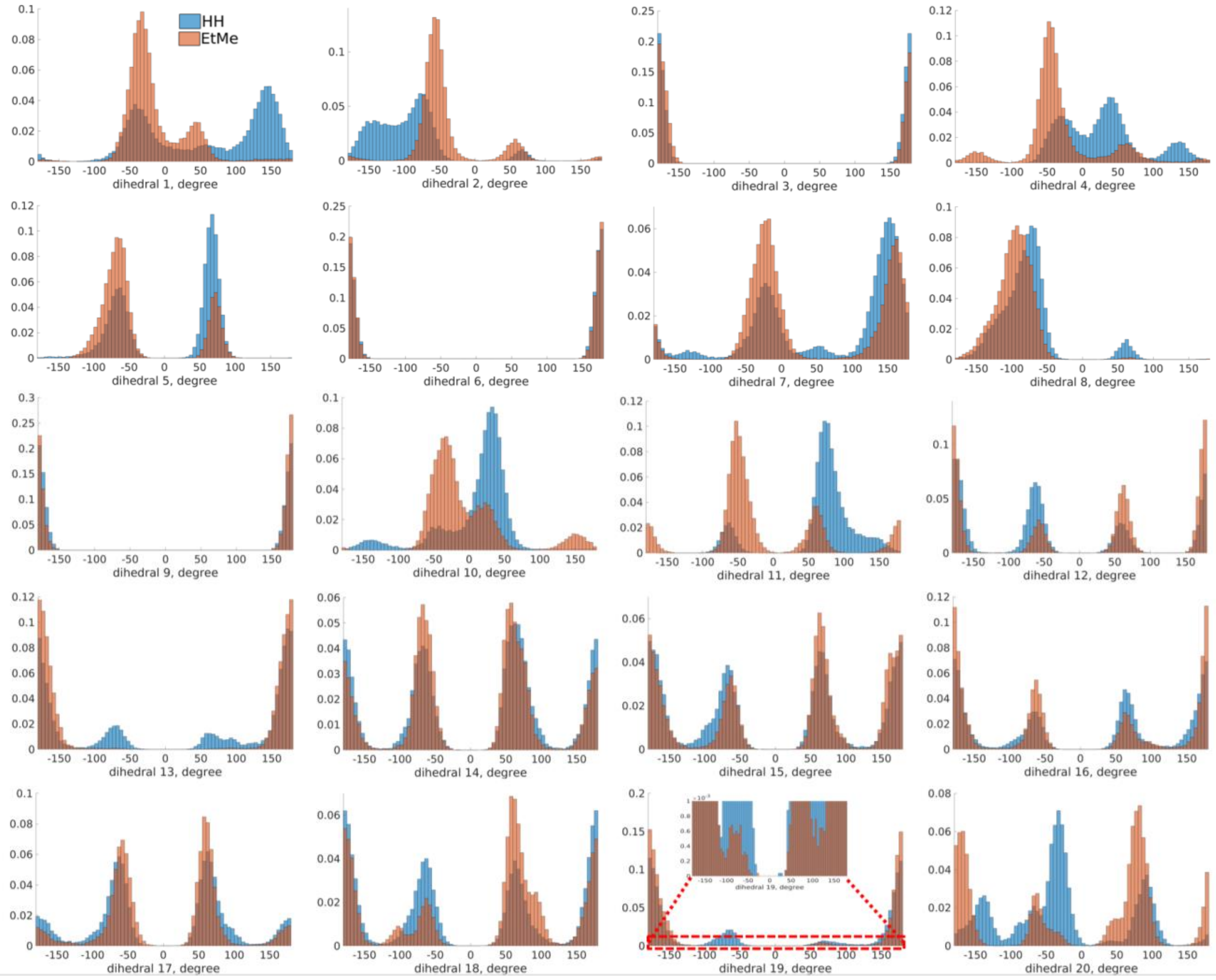

Figure S20: Distributions of dihedral angles in the macrocycle as defined in Figure S19 for **9** (H/H) an **11** (Et/Me) ligands. As expected one can see a clear difference in the distributions between ligand **9** (H/H) an **11** (Et/Me) for most of the dihedral angles at the alkylation sites (dihedrals 1, 2, 10, 11, 20). For the "other" dihedral angles the peaks positions on the distributions remain approximately the same, but the height of the peaks differs in some cases. This illustrates that the conformational restriction introduced by alkyl substituents is propagated to other dihedral angles of the macrocycle. The distributions of the "other" angles indicate that for both ligands similar conformational space was available for sampling in the REST MD simulations, since for every peak we observe some (sometimes negligible) population for both ligands (e.g. see the zoom in plot for dihedral 19). From this analysis we conclude that the sampling of macrocycle dihedral angles in the REST MD simulations was adequate.

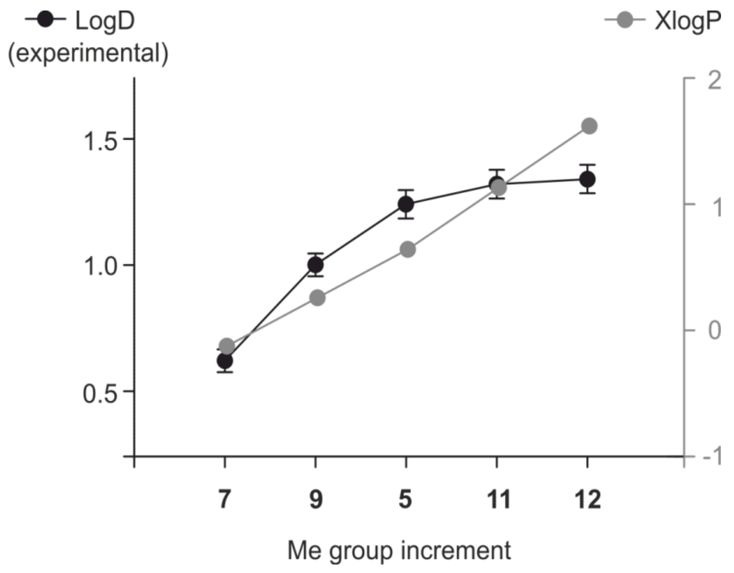

Figure S21: Experimentally determined LogD-values (black) and calculated XlogP-values (grey) of **7** (H/H), **9** (Me/H), **5** (Me/Me), **11** (Et/Me) and **12** (Et/Et) as a function of methylene group increment. Experimental LogD data show nonlinear dependence, whereas XlogP-values and methylene group increment reveal an almost linear correlation.

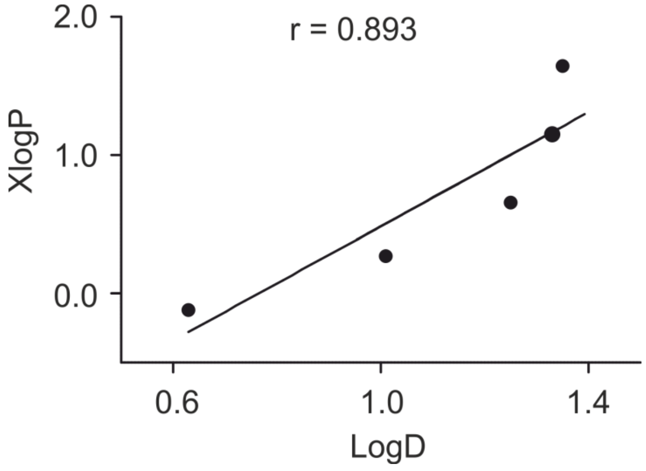

Figure S22: Calculated LogP using a conformation agnostic approach (2D, XlogP from OpenEye) versus measured LogD. Linear regression line shown in black (Pearson correlation coefficient r = 0.893).

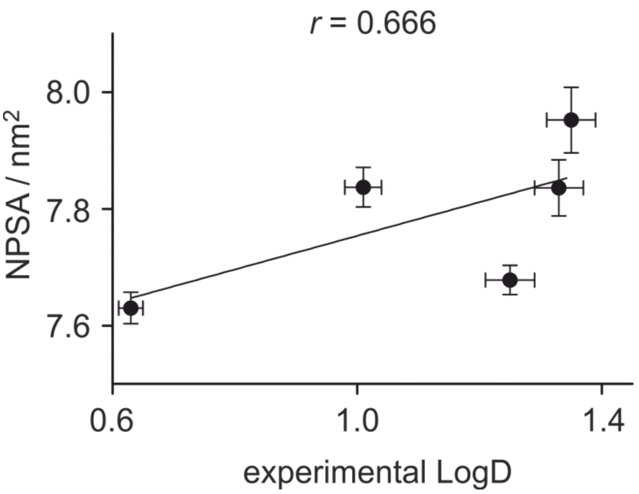

Figure S23: Dynamic NPSA derived from REST MD simulation versus measured LogD, linear regression line shown in black (Pearson correlation coefficient r = 0.666).

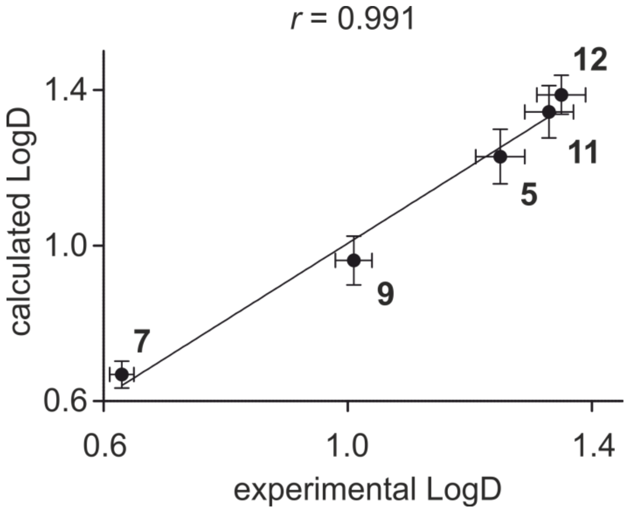

Figure S24: Multi-linear regression of calculated LogD based on dynamic PSA and NPSA descriptors (LogD = 0.27 · NPSA – 0.82 · PSA + 2.22) versus measured LogD providing an excellent correlation between calculated and experimental LogD-values. The linear regression line shown in black (Pearson correlation coefficient r = 0.991).

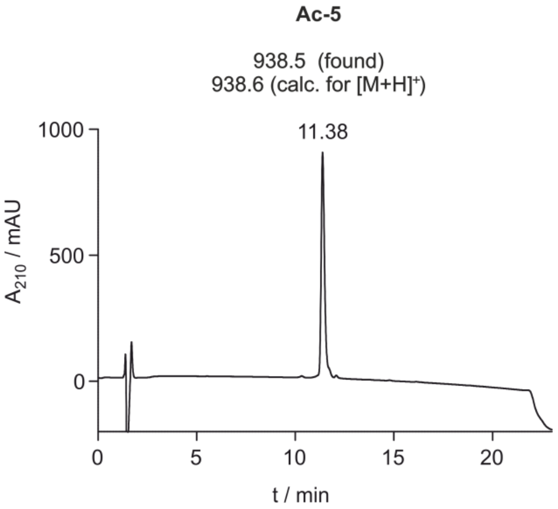

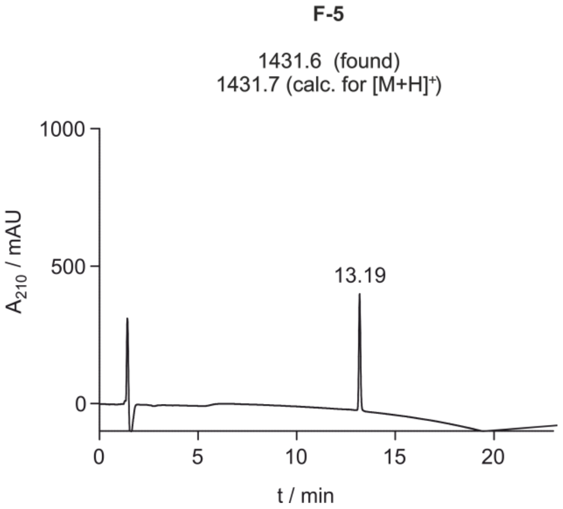

Figure S25: HPLC chromatogram detected at λ = 210 nm for peptides **5** (Me/Me) with *N*-terminal acetyl- (left) and FITC-peg$_2$- (right) label, respectively, including peak retention time t_R and corresponding found and calculated $[M+H]^+$-values.

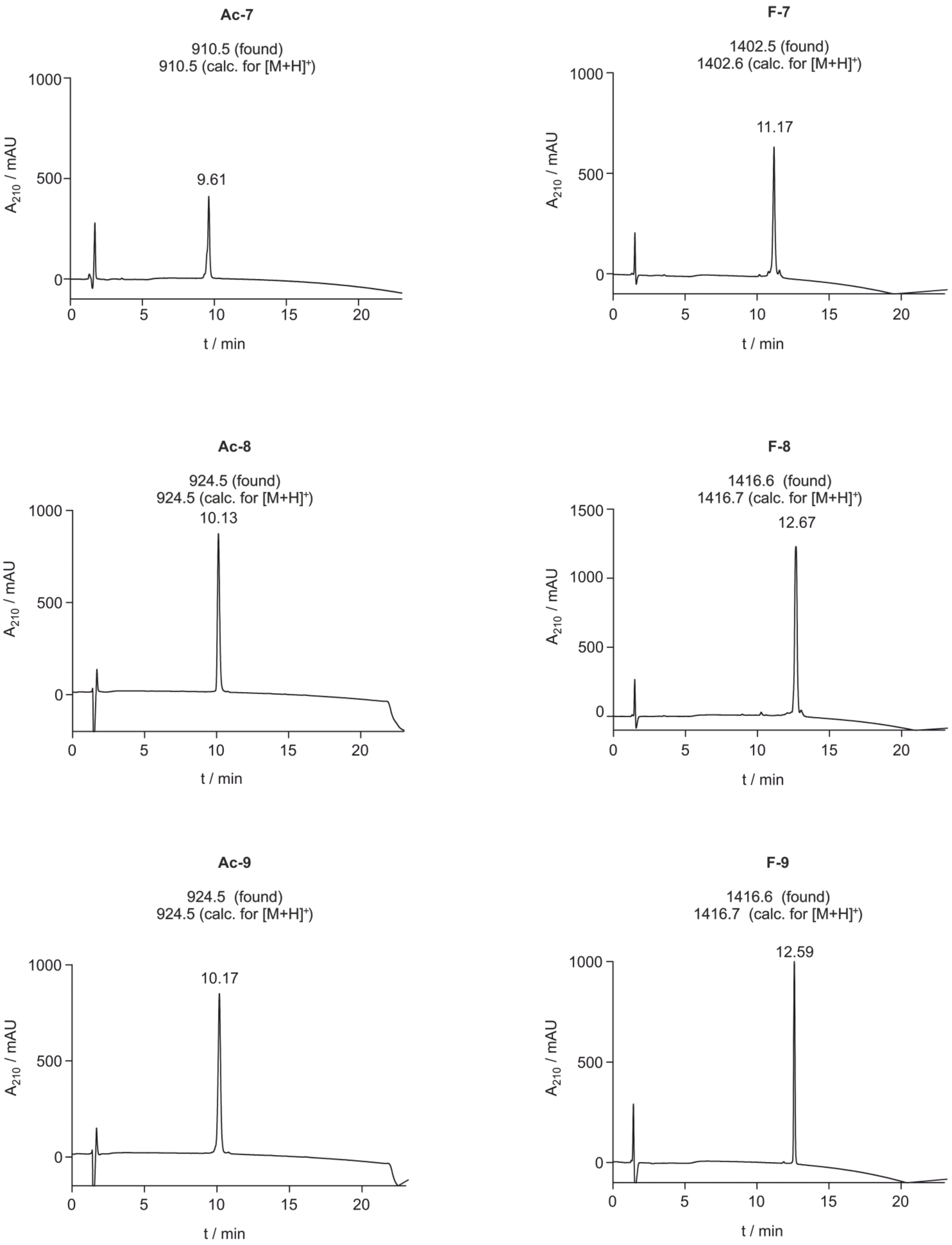

Figure S26: HPLC chromatogram detected at λ = 210 nm for hydrogen modified peptides **7** – **9** with *N*-terminal acetyl- (left) and FITC-peg$_2$- (right) label, respectively, including peak retention time t_R and corresponding found and calculated $[M+H]^+$-values.

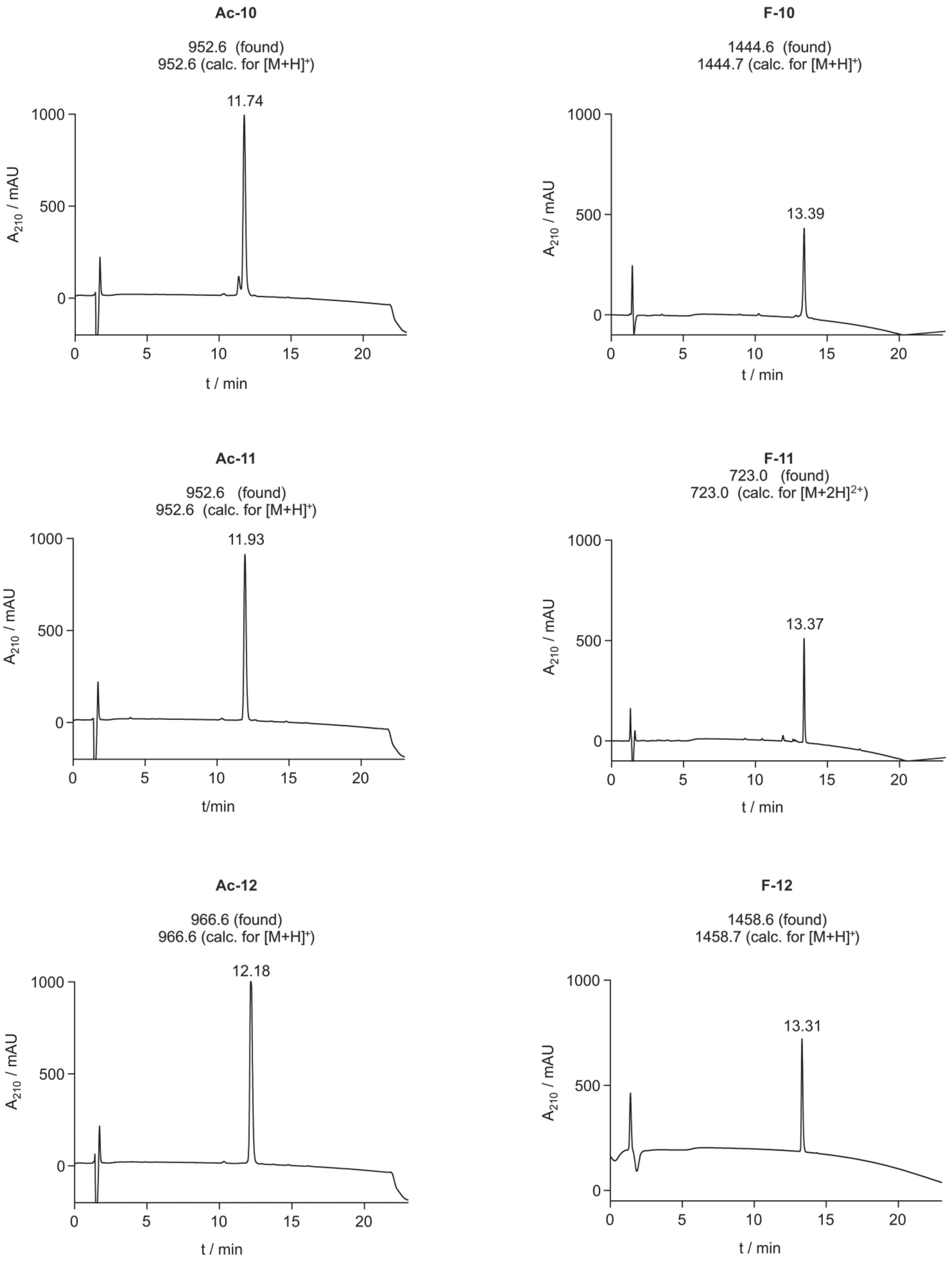

Figure S27: HPLC chromatogram detected at λ = 210 nm for ethyl modified peptides **10** – **12** with *N*-terminal acetyl- (left) and FITC-peg$_2$- (right) label, respectively, including peak retention time t_R and corresponding found and calculated $[M+H]^+$ and $[M+2H]^{2+}$-values.

4. References

1 G. H. Bird, W. Christian Crannell and L. D. Walensky, *Curr. Protoc. Chem. Biol.*, 2011, **3**, 99–117.

2 Z. Nikolovska-Coleska, R. Wang, X. Fang, H. Pan, Y. Tomita, P. Li, P. P. Roller, K. Krajewski, N. G. Saito, J. A. Stuckey and S. Wang, *Anal. Biochem.*, 2004, **332**, 261–273.

3 E. H. Kerns, L. Di, S. Petusky, T. Kleintop, D. Huryn, O. McConnell and G. Carter, *J. Chromatogr. B Anal. Technol. Biomed. Life Sci.*, 2003, **791**, 381–388.

4 M. H. Abraham, F. Gao, M. Y. Shalaeva, K. A. Tupper and F. Lombardo, *J. Med. Chem.*, 2002, **43**, 2922–2928.

5 Y. C. Martin, *J. Med. Chem.*, 2002, **39**, 1189–1190.

6 D. Shivakumar, E. Harder, W. Damm, R. A. Friesner and W. Sherman, *J. Chem. Theory Comput.*, 2012, **8**, 2553–2558.

7 J. Berendsen, H.J.C., Postma, J.P.M., van Gunsteren, W.F. and Hermans, in *Intermolecular Forces*, 1981, pp. 331–342.

8 L. Wang, Y. Wu, Y. Deng, B. Kim, L. Pierce, G. Krilov, D. Lupyan, S. Robinson, M. K. Dahlgren, J. Greenwood, D. L. Romero, C. Masse, J. L. Knight, T. Steinbrecher, T. Beuming, W. Damm, E. Harder, W. Sherman, M. Brewer, R. Wester, M. Murcko, L. Frye, R. Farid, T. Lin, D. L. Mobley, W. L. Jorgensen, B. J. Berne, R. A. Friesner and R. Abel, *J. Am. Chem. Soc.*, 2015, **137**, 2695–2703.

9 L. Wang, Y. Deng, J. L. Knight, Y. Wu, B. Kim, W. Sherman, J. C. Shelley, T. Lin and R. Abel, *J. Chem. Theory Comput.*, 2013, **9**, 1282–1293.

10 C. H. Bennett, *J. Comput. Phys.*, 1976, **22**, 245–268.

11 M. J. Abraham, T. Murtola, R. Schulz, S. Páll, J. C. Smith, B. Hess and E. Lindah, *SoftwareX*, 2015, **1**–**2**, 19–25.

12 A. I. Frolov and M. G. Kiselev, *J. Phys. Chem. B*, 2014, **118**, 11769–11780.

13 F. Eisenhaber, P. Lijnzaad, P. Argos, C. Sander and M. Scharf, *J. Comput. Chem.*, 1995, **16**, 273–284.

14 A. Ben-Naim, *Solvation Thermodynamics*, 1987.
